# The Role of Introgression During the Radiation of Endemic Fishes Adapted to Living at Extreme Altitudes in the Tibetan Plateau

**DOI:** 10.1093/molbev/msad129

**Published:** 2023-05-29

**Authors:** Yuting Qian, Minghui Meng, Chaowei Zhou, Haiping Liu, Haifeng Jiang, Youwei Xu, Wenjun Chen, Zufa Ding, Yang Liu, Xiong Gong, Cheng Wang, Yi Lei, Tai Wang, Ying Wang, Xiaoni Gan, Axel Meyer, Shunping He, Liandong Yang

**Affiliations:** State Key Laboratory of Freshwater Ecology and Biotechnology, Institute of Hydrobiology, Chinese Academy of Sciences, Wuhan, China; State Key Laboratory of Plateau Ecology and Agriculture, Qinghai University, Xining, China; Academy of Plateau Science and Sustainability, Qinghai Normal University, Xining, China; University of Chinese Academy of Sciences, Beijing, China; Key Laboratory of Horticultural Plant Biology (MOE), College of Horticulture and Forestry Sciences, Huazhong Agricultural University, Wuhan, China; College of Fisheries, Southwest University, Chongqing, China; Key Laboratory of Freshwater Fish Reproduction and Development, Ministry of Education, Key Laboratory of Aquatics Science of Chongqing, Chongqing, China; Integrative Science Center of Germplasm Creation in Western China (CHONGQING) Science City, Key Laboratory of Freshwater Fish Reproduction and Development (Ministry of Education), Key Laboratory of Aquatic Science of Chongqing, School of Life Sciences, Southwest University, Chongqing, China; College of Animal Science and Technology, Northwest A&F University, Yangling, Shaanxi, China; South China Sea Fisheries Research Institute, Chinese Academy of Fishery Sciences, Guangzhou, China; State Key Laboratory of Freshwater Ecology and Biotechnology, Institute of Hydrobiology, Chinese Academy of Sciences, Wuhan, China; University of Chinese Academy of Sciences, Beijing, China; State Key Laboratory of Freshwater Ecology and Biotechnology, Institute of Hydrobiology, Chinese Academy of Sciences, Wuhan, China; State Key Laboratory of Freshwater Ecology and Biotechnology, Institute of Hydrobiology, Chinese Academy of Sciences, Wuhan, China; State Key Laboratory of Freshwater Ecology and Biotechnology, Institute of Hydrobiology, Chinese Academy of Sciences, Wuhan, China; University of Chinese Academy of Sciences, Beijing, China; State Key Laboratory of Freshwater Ecology and Biotechnology, Institute of Hydrobiology, Chinese Academy of Sciences, Wuhan, China; University of Chinese Academy of Sciences, Beijing, China; State Key Laboratory of Freshwater Ecology and Biotechnology, Institute of Hydrobiology, Chinese Academy of Sciences, Wuhan, China; Gansu Key Laboratory of Cold Water Fishes Germplasm Resources and Genetics Breeding, Gansu Fisheries Research Institute, Lanzhou, China; Academy of Plateau Science and Sustainability, Qinghai Normal University, Xining, China; Hubei Engineering Research Center for Protection and Utilization of Special Biological Resources in the Hanjiang River Basin, School of Life Sciences, Jianghan University, Wuhan, China; State Key Laboratory of Freshwater Ecology and Biotechnology, Institute of Hydrobiology, Chinese Academy of Sciences, Wuhan, China; Department of Biology, University of Konstanz, Konstanz, Germany; State Key Laboratory of Freshwater Ecology and Biotechnology, Institute of Hydrobiology, Chinese Academy of Sciences, Wuhan, China; Academy of Plateau Science and Sustainability, Qinghai Normal University, Xining, China; Center for Excellence in Animal Evolution and Genetics, Chinese Academy of Sciences, Kunming, China; State Key Laboratory of Freshwater Ecology and Biotechnology, Institute of Hydrobiology, Chinese Academy of Sciences, Wuhan, China; State Key Laboratory of Plateau Ecology and Agriculture, Qinghai University, Xining, China; Academy of Plateau Science and Sustainability, Qinghai Normal University, Xining, China

**Keywords:** phylogenetic conflict, introgression, reticulation, recombination, *Triplophysa*

## Abstract

Recent genomic analyses of evolutionary radiations suggest that ancient introgression may facilitate rapid diversification and adaptive radiation. The loach genus *Triplophysa*, a genus with most species endemic to Tibetan Plateau, shows ecological diversity and rapid evolution and represents a potential example of adaptive radiation linked to the uplift of the Tibetan Plateau. Here, we interrogate the complex evolutionary history of *Triplophysa* fishes through the analysis of whole-genome sequences. By reconstructing the phylogeny of *Triplophysa*, quantifying introgression across this clade, and simulating speciation and migration processes, we confirm that extensive gene flow events occurred across disparate *Triplophysa* species. Our results suggest that introgression plays a more substantial role than incomplete lineage sorting in underpinning phylogenetic discordance in *Triplophysa*. The results also indicate that genomic regions affected by ancient gene flow exhibit characteristics of lower recombination rates and nucleotide diversity and may associate with selection. Simulation analysis of *Triplophysa tibetana* suggests that the species may have been affected by the Gonghe Movement in the third uplift of the Tibetan Plateau, resulting in founder effects and a subsequent reduction in Ne.

## Introduction

Studies of phenotypic diversification and phylogenetic discordance have been foundational to our understanding of evolution. One classic example of these two phenomena is reticulate evolution during adaptive radiation (Mallet et al. 2016; Jamie and Meier 2020). Genomic analyses have shown that phylogenies of closely related species often show a high degree of reticulation, which is often associated with adaptive radiation (Fontaine et al. 2015; Meier et al. 2017; Edelman et al. 2019; Choi et al. 2021; Hu et al. 2022). Ancestral variation and subsequent hybridization generate the initial diversity that can then be sculpted by selection into an adaptive radiation (Berner and Salzburger 2015; Meier et al. 2017; Choi et al. 2021; Nelson et al. 2021). The potential for rapid adaptation to new environments and range expansion via adaptive introgression may increase with increasing amounts of genetic polymorphisms (Hedrick 2013).

Genetic exchange between lineages is generally assumed to hinder the evolution of reproductive isolation (Kulmuni et al. 2020), yet genetic exchange between lineages can also be a persistent feature of diversification of a clade and obscure the relationships among ancient branches in a phylogenetic tree (Irisarri et al. 2018; Yang et al. 2021). A phylogeny is used to visualize the evolutionary history of a set of taxa. However, phylogenetic discordance is widespread in phylogenetic analysis (Prum et al. 2015; Irisarri et al. 2017; Leebens-Mack et al. 2019), and the causes of this are manifold, difficult to characterize, and not always due to hybridization. Conflicting patterns between mitochondrial and nuclear genetic markers have been considered to be related to (sometimes adaptive), for example, introgression of mitochondrial DNA (mtDNA), demographic disparities, and sex-biased asymmetries (Toews and Brelsford 2012; Kang et al. 2013). Discordance between gene trees and species trees can also be caused by incomplete lineage sorting (ILS) or other phenomena such as horizontal gene transfers (Nichols 2001; Ruber et al. 2001; Avise and Robinson 2008; Solis-Lemus and Ane 2016). Some phylogenetic relationships are simply difficult to resolve because they involve ancient splits, with weak phylogenetic signal that occurred in short succession (Irisarri and Meyer 2016; Irisarri et al. 2017). Even the concatenation of extensive sequence data sets to analyze phylogenetic relationships in cases where lineages show frequent introgression may be insufficient for robust phylogenetic inference (Kubatko and Degnan 2007; Shen et al. 2021). Species trees can be constructed using the multispecies coalescent-based approach (Rannala and Yang 2003), but this approach might be affected by low-quality individual gene alignments to infer individual gene trees (Blom et al. 2017; Simmons and Kessenich 2020).

If introgression affects large parts of the genome, inferred phylogenies will be affected by this history of reticulate evolution in a given lineage (Mallet et al. 2016). Analyzing complete genomes in an effort to examine adaptive radiations might allow to assess the role of ancestral genetic variation in speciation and phenotypic diversification (Meier et al. 2017; Svardal et al. 2020; Wang, Street, et al. 2020). The combination of natural selection and recombination can produce heterogeneous patterns of genomic divergence between nascent and recently evolved species (Meyer et al. 2006; Via and West 2008; Nosil et al. 2009). On one hand, genomic regions of low recombination linked with deleterious loci will be broken and purged, preventing the retention of traces of introgression suitable as a data source for reconstructing phylogenetic relationships(Schumer et al. 2018; Edelman et al. 2019; Li et al. 2019; Martin et al. 2019; Nelson et al. 2021). On the other hand, genomic regions that have undergone hybridization, introgression, and subsequent selection may make it difficult to accurate estimate species tree (Nater et al. 2015; Zhang et al. 2021).

The Tibetan Plateau is the youngest and highest plateau on Earth with the height average above 4,500 m sea level. As it uplifted, its geographical features and natural environment have experienced drastic change (Deng and Ding 2015; Su et al. 2019). *Triplophysa* is a genus of nemacheiline loaches (family Cyprinidae), the most species-rich lineage of freshwater fishes in Asia. *Triplophysa* currently includes about 140 species that can be found at altitudes up to 5,200 m. Hence, it is widely distributed across and span the entire Tibetan Plateau and adjacent regions in Central Asia (He et al. 2011; Wang et al. 2015; Li et al. 2017; Wang, Zhang, et al. 2020). This genus is variable in coloration and morphology, with different patches or spots on the body surface, and different anatomical features (Yang, Liu, et al. 2019; Yang, Wang, et al. 2019; Wang, Zhang, et al. 2020; Yuan et al. 2020; Zhou et al. 2021). They usually inhabit still waters or slow-flowing bottoms of rivers and lakes, feeding on aquatic invertebrates or algae (Bureau of fisheries of Tibet Autonomous Region 1995). Previous studies based on morphological and biogeographic data have found that changes in geography and climate associated with the uplifting of the Tibetan Plateau were, through allopatric speciation, the driving forces behind the formation of the species diversity of *Triplophysa* fishes (He et al. 2006). The main river systems in the Tibetan Plateau region–the Hexi River system, the upper reaches of the Huanghe River, and the Jialing River not only share several species of *Triplophysa* but also possess endemics (Wang and Chang 2012; Wang, Zhang, et al. 2020). Divergent populations of *Triplophysa* may come into secondary contact due to geological changes such as mountain upheaval and river diversion (Fang et al. 2005; Favre et al. 2015), resulting in genetic exchange (Feng et al. 2019).

Here, we use whole-genome sequences (WGS) of 18 representative species spanning all major lineages of the radiation of *Triplophysa* to explore the phylogenetic relationships between them and to gain deeper insights into the evolutionary processes that shaped it and characterized their genomic architectures. In this study, we estimated a comprehensive phylogenetic relationship of the genus *Triplophysa*, then quantified signals of introgression, and simulated the history of speciation and migration events. We show the abundance of allele sharing across this clade, which may have played a crucial role during the adaptive radiation of *Triplophysa* fishes. We also find evidence of an ancient gene flow event occurred ∼0.24–9.87 million years ago (Ma) between *Triplophysa tibetana* and a clade of *Triplophysa* fishes. Our analyses show that genomic regions under introgression reveal a characteristic of lower recombination rates and nucleotide diversity that have similarities with regions under selection. This is a tentatively exploration of genome-wide patterns of introgression and may help us understand the driving force behind the adaptation of *Triplophysa* fishes to the Tibetan Plateau.

## Results

### Summary of Sequencing, Mapping, SNP Calling, and Filtering

A total of 138 *Triplophysa* from 18 species were whole-genome resequenced on an Illumina platform, which contains the major lineages of *Triplophysa* species that have been reported in Northwest China (He et al. 2011). Most were collected from the northeastern edge of the Tibetan Plateau (Gansu Province), and some samples of *Triplophysa orientalis*, *Triplophysa bleekeri*, *Triplophysa stewarti*, and *T. tibetana* were collected from Lhasa (Xizang Autonomous Region) (supplementary table S2, Supplementary Material online). After quality control, ∼3.91 Tb of whole-genome sequencing data (26.2 billion paired-end clean reads) were generated for 138 *Triplophysa* samples at an average depth of ∼34.7× per sample. The mapping rate of these clean reads to the *T. tibetana* reference genome was 96.10% ± 1.83%, and the genome coverage was >80% across all chromosomes for the majority of samples (supplementary table S1, Supplementary Material online). Sequence reads were aligned to the *T. tibetana* reference genome (Yang, Liu et al. 2019) and variants called, generating a total of 11.6 million single-nucleotide variants (SNVs).

### Population Structure and Genetic Differentiation Among the Species

**Fig. 1.**
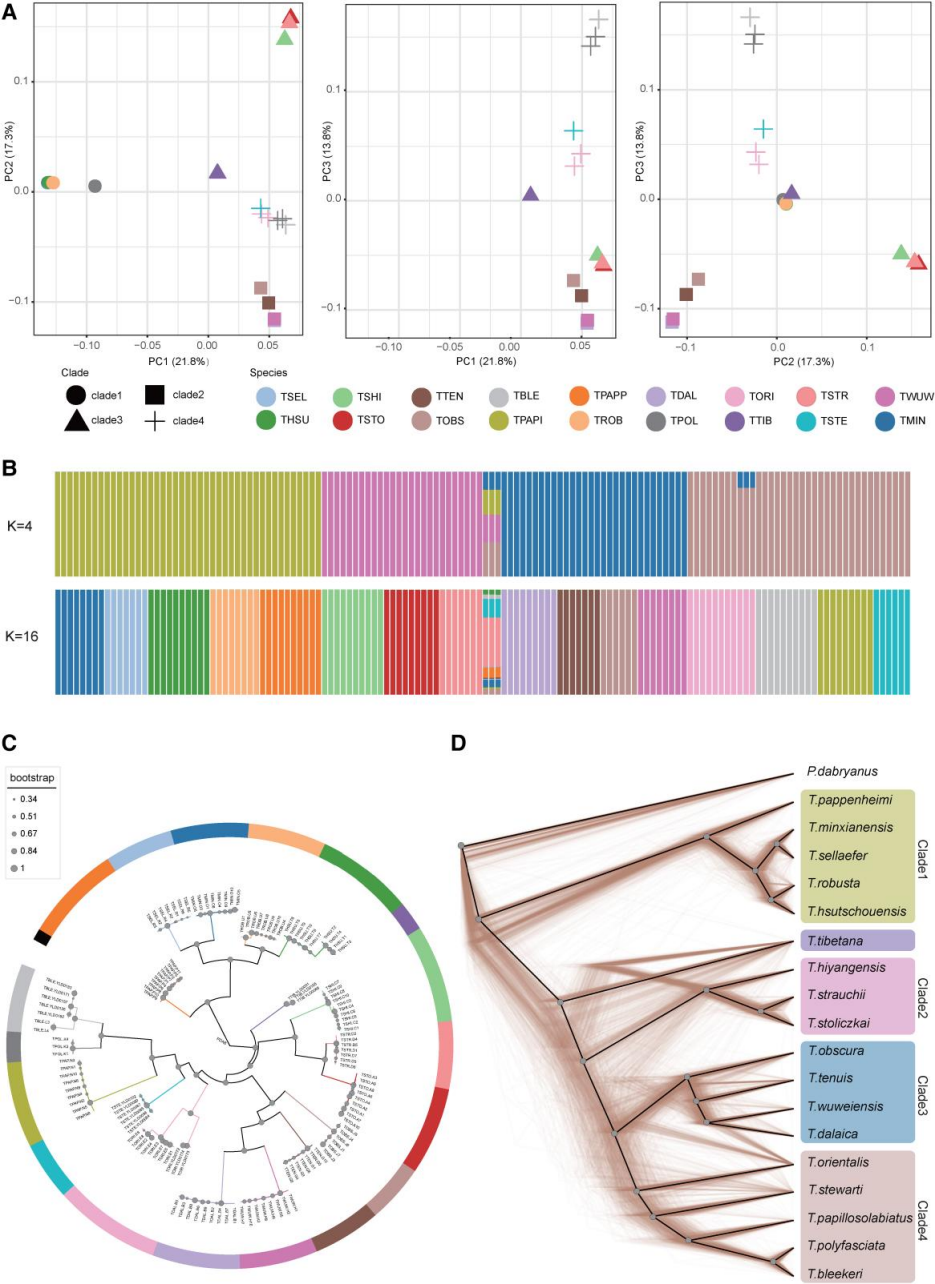
Population structure and inferred phylogenetic relationships among *Triplophysa* species. (*A*) PCA of 138 *Triplophysa* individuals. (*B*) Population structure by STRUCTURE analysis assign individuals to five clusters with *K* = 4, and the optimal *K* = 16, which can assign most individuals to well-defined species. Each colored bar represents one individual, and colored segments represent proportions of genetic components. (*C*) The coalescent-based species tree. The node size represents ASTRAL LPP according to the scale shown on the left. The color of the branches refers to the species as in *A* and *B* (color scheme as in *B*, *K* = 16, except *T. tibetana* and *T. polyfasciata*). (*D*) Cladograms of the prevalent discordances trees among 1,000 randomly selected local trees derived from 200-kb windows, and solid black line represents the consensus tree calculated by DensiTree, which is consistent with Astral species tree (color scheme as in *B*, *K* = 4).

We first described the patterns of genome-wide diversity using principal component analysis (PCA), which revealed a clear distinction among *T. tibetana* clade and grouped all individuals into four or five genetic clusters (fig. 1*A*). The first three principal components (PC1, PC2, and PC3; explained 21.8%, 17.3%, and 13.8%) separated *T. tibetana* (TTIB) from the other four clusters, but some *T. orientalis* (TORI) and *T. bleekeri* (TBLE) collected from the adjacent area, Lhasa, were closer to *T. tibetana*. It is worth noting that in all three principal components, *T. tibetana* were placed between to the main clusters, and many processes can lead to patterns of genetic intermediacy we observed, including hybridization (Liang et al. 2019; Liu et al. 2022).

We then estimated individual genetic assignment to detect ancient admixture or recent introgression. In the admixture plot at *K* = 4 (fig. 1*B*), the categories of population were consisted with major cluster in consensus with PCA analysis. At *K* = 5, *T. orientalis* and *T. tibetana* were divided into new genetic clusters. At *K* ≤ 16 (supplementary fig. S2, Supplementary Material online), all clusters did not reveal evidence for strong admixture between species, except *T. tibetana* and three individuals of *T. orientalis*. This result suggests a pattern of genetic heterogeneity and could be caused by insufficient sample size in structure analysis (Dupuis and Sperling 2020; Hinojosa et al. 2022). The optimal *K* value is *K* = 16 (cross-validation error 0.21; supplementary fig. S3, Supplementary Material online), which grouped all individuals by species except *T. tibetana* and the cluster of *Triplophysa polyfasciata* and *T. bleekeri*. Some individuals of those two species were sampled from adjacent geographic areas, which may be the reason for this population structure.

Out-groupf3 tests were used to measure the genetic relationships between all sampled *Triplophysa* species. The heat map of f3 estimated values among pairs of species (supplementary fig. S4, Supplementary Material online) shows a similar pattern as the PCA results. *T. tibetana* had the highest genetic affinities to clade2, clade3, and clade4, particularly clade2 and *T. stewarti*. These results indicate that *T. tibetana* might have had genetic contact with these three clades after its initial divergence.

### Phylogenetic Relationships Between the Species

Based on the nuclear genomic level data set, we used concatenation and multispecies coalescent approaches to further test the robustness of the inferred phylogenetic relationships between *Triplophysa* fishes. Five data sets from nuclear genomic sliding windows generated with partition strategies of nonoverlapping windows of 200, 100, 50, 25, and 10 kb and one data set of binned genes were used to infer local phylogenies for each window or gene. Then, we generated coalescent-based ASTRAL species trees with local trees or gene trees to reconstruct the consensus tree for each data set. We also concatenated all filtered single-nucleotide polymorphism (SNP) sites across the WGS and only protein coding sequences (CDS) and 4-fold degenerate (4D) sites to generate three concatenated data sets. Finally, we constructed the concatenation-based phylogenetic tree (supplementary fig. S5, Supplementary Material online).

All phylogenies generated from these two approaches were supported with high local posterior probability (LPP in ASTRAL) or bootstrap values (supplementary table S3, Supplementary Material online). Five species trees based on coalescent method and local tree data sets result in the identical phylogeny (fig. 1*C*), and the gene tree data sets supported a different phylogenetic structure. The concatenation-based approach reconstructed two different phylogenetic relationships. The phylogenies of the CDS data set and the 4D data set are consistent with each other and the gene tree ASTRAL topology. The only discordant feature among all phylogenies was the position of *T. tibetana* (supplementary fig. S5, Supplementary Material online). Based on the distance from the outgroup, all four major clades in the phylogenetic tree could be clearly identified, except for the placement of *T. tibetana*. All five ASTRAL species trees supported a monophyletic clade formed by clade2, clade3, and clade4 as the sister to *T. tibetana*. Whereas the WGS tree supported a monophyletic clade clustered by clade1, clade2, clade3, and clade4 as sister to *T. tibetana*, the CDS/4D tree supported *T. tibetana* as sister to clade2.

### Alternative Tree Topologies From Coalescent-Based Analysis

We explored the phylogenetic relationships of the *Triplophysa* species using TWISST (Martin and Van Belleghem 2017), which quantifies the frequency of alternative phylogenetic topologies in sliding windows along the genome. Our coalescent-based phylogenetic method inferred two kinds of species tree from local and gene tree sets. After merging clade3 and clade4 into one clade, the two topologies can be expressed as Topo A: (PDAB, (clade1, (TTIB, (clade2, clade3-4)))); and Topo B: (PDAB, (clade1, (clade3-, (clade2, TTIB)))); PDAB represents outgroup *Paramisgurnus dabryanus*. The most common topologies in sliding window trees and gene trees were also consistent with those two topologies, respectively (supplementary fig. S6*a*–*f*, Supplementary Material online). The ASTRAL species tree based on the normalized quartet score of 5 sliding windows reveals a positive correlation with window size (supplementary table S4, Supplementary Material online): As the window size decreased, the proportion of all the quartet trees that could be found in the species tree also decreased. Topology weighting analysis of window local maximum likelihood (ML) trees reveals that, in about 44.5–51.1% local trees, *T. tibetana* is sister to a cluster formed by clade2, clade3, and clade4 (Topo A) and, in about 37.6–43.5% local trees, *T. tibetana* and clade2 formed a cluster (Topo B) (supplementary fig. S6, Supplementary Material online). Topological discordance can also be revealed from the DensiTree plot of 1,000 random 200-kb local trees (fig. 1*D*). In addition to the discordant placement of *T. tibetana*, there are also different topologies within each major clade, which indicates a high degree of topological uncertainty (Bouckaert 2010). The different result was presented in topology weighting analysis of gene trees, which shows >50% of trees support Topo B and only 46% support Topo A. This discordance exemplifies that gene trees may differ from the species tree due to ILS or admixture between lineages and also reveals correlations among different data set sources used to construct phylogenetic trees.

### Evidence for Rampant Past Introgression

**Fig. 2.**
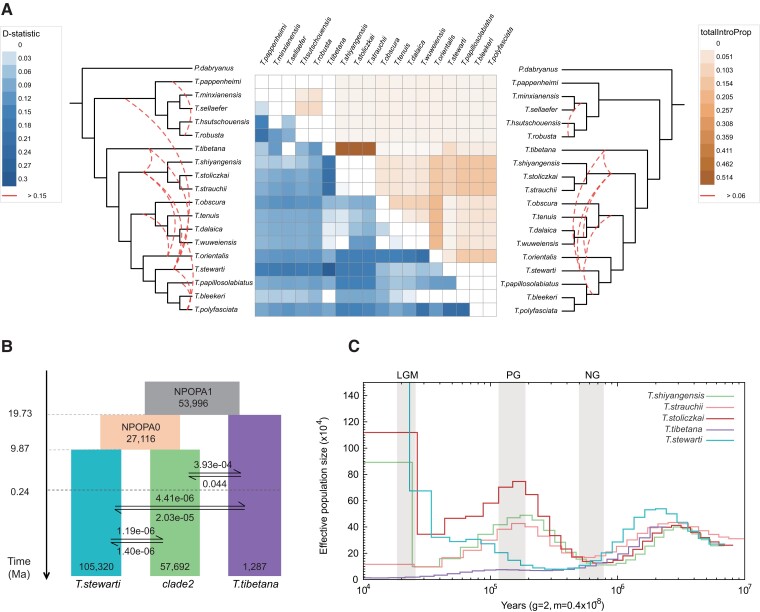
Introgression between *Triplophysa* species. (*A*) Heat map of average pairwise *D* values per species pair (in left) and the average totalIntroProp per species pair inferred through the QuIBL analysis (in right). Empty squares represent zero or nonsignificant values. The species tree is shown on both sides. Red dotted lines on the species tree of two sides indicate interspecific introgression events based on summarized results with threshold of *D* > 0.15 and totalIntroProp > 0.06, respectively. (*B*) Schematic of demographic history of the three *Triplophysa* species simulated by fastsimcoal2. Rectangles represent each species population and their ancestral lineages. The numbers on the bottom of rectangle represent Ne, and arrows indicate the per-generation migration rate (*m*) between species populations. The time node of each historical scenario is marked on the left timeline. (*C*) Demographic history inferred by the PSMC model. Lines represent the median estimated Ne of five *Triplophysa* species. The periods of the LGM, PG, and NG are highlighted with gray vertical bars.

In addition to ILS, phylogenetic conflict between genomic partitions may also be explained by hybridization (Cahill et al. 2013; Li et al. 2016). When interbreeding is not restricted between immigrant and local species, introgression in neutral sites can take place on a wide genomic or geographic scale (Currat et al. 2008). To test whether the speciation process among *Triplophysa* was accompanied by past introgression, we calculated Patterson's *D*-statistic using ADMIXTOOLS (Patterson et al. 2012), which assesses the extent of genetic admixture among a quartet of species populations. A total of 816 quartets conformed to the species tree topology were detected (supplementary table S8, Supplementary Material online), and 100% of the quartets reached a threshold of |Z| > 3, which indicates a significant *D* value. In quartet topologies of the form (W, X, Y, OUT), W, X, and Y represent one species population from clade1, clade2, clade3, clade4, and *T. tibetana* in the species tree. We classified the triplets (W, X, Y) into species pairs, based on positive and negative values of the *D* value. Thus, we calculated mean D-values for species pairs using W and Y as a species pair if the D-value for the triplet was positive, and X and Y as a species pair if the D-value for the triplet was negative. We visualized the 136 pairs of D-value averages with a heat map (fig. 2*A*). The largest *D* value appears in the species pair of TTIB_TSTE (*T. stewarti*), which indicated a high level of genetic introgression between *T. stewarti* and *T. tibetana*. Following are the TTIB_TSTR (*Triplophysa strauchii*), TTIB_TSTO (*Triplophysa stoliczkai*), and TTIB_TSHI (*Triplophysa shiyangensis*) pairs, which feature species clustered in clade2. Similar *D* values were found between clade2 and *T. tibetana*, which suggests an episode of ancient gene flow between *T. tibetana* and the ancestral lineage of clade2. *T. stewarti* also experienced a significant gene flow event between itself and clade1/clade2 supported by high *D*-statistic values. We also detected significant gene flow between *T. orientalis* and all species in clade2/clade3, with high *D* values. Considering the chronological order of speciation (supplementary fig. S7, Supplementary Material online), we speculate that the gene flow event occurred between *T. orientalis* and the ancestral lineage of two clades. The species pairs showing incongruence across the phylogenies were supported by significant *D* values (fig. 1*D*), which suggests that gene flow might have contributed to the observed phylogenetic discordance.

To further distinguish whether the observed phylogenetic discordance were indeed due to introgression rather than ILS, we conducted gene tree set analysis in QuIBL (Edelman et al. 2019), which estimates observed distributions of internal branch lengths for triplet topologies. Among 816 triplets, we only found one triplet that showed a significant level of ILS (supplementary table S9, Supplementary Material online). Another 413 triplets showed significant levels of introgression (*Δ*BIC > 10), accounting for 50.6% of the total triplet sets (supplementary table S9, Supplementary Material online). We also divided all the results into 132 species pairs and calculated the average of the total introgression proportion (totalIntroProp) for each species pair. We then visualized the 132 pairs of totalIntroProp averages in a heat map (fig. 2*A*) and marked introgression on the species tree with a red dotted line for the species pair of totalIntroProp > 0.06. Most high value results were consistent with the *D*-statistic analyses. Introgression signatures between *T. tibetana*–clade2, *T. tibetana*–clade4, *T. orientalis*–clade2, *T. orientalis–T. tibetana*, and *T. stewarti*–clade2 emerged. Hence, across the two analytical results, we found evidence for extensive introgression among several *Triplophysa* species, suggesting phylogenetic uncertainties caused by past hybridization between lineages rather than ILS.

### Demographic History Infers Ancient Introgression

We performed coalescent-based demographic simulations using fastsimcoal2.7 (Excoffier et al. 2013), to complement details of introgression between *Triplophysa* species. We found that model7 (fig. 2*B*) presented higher likelihood values and lowest Akaike information criterion (AIC) weights than other models (supplementary table S5, Supplementary Material online). The simulation results estimated divergence events TDIV1, TDIV0 at 9,863,719 generations (equating to 19.73 Ma) and 4,934,948 generations (9.87 Ma) ago, respectively, and also suggested an ancient episode of gene flow between *T. tibetana* and clade2 at 122,301–4,934,948 generations (0.24–9.87 Ma). This species pair had a higher estimated migration rate (0.045/3.92e−04) than other species pairs, which agrees with the previous *D*-statistic and QuIBL analyses. Gene flow between the other two pairs of species *T. tibetana*–*T. stewarti* and *T. stewarti*–clade2 occurred after 122,301 generations (0.24 Ma). The assumptions that introgression across those three species pairs occurred after *T. stewarti* diverged and continues to the present (supplementary fig. S8*b*, Supplementary Material online), and all introgression events occurred in ancient ages (supplementary fig. S8*h*, Supplementary Material online) were not supported.

We used a pairwise sequential Markovian coalescent (PSMC) model to infer the demographic histories of the five *Triplophysa* species that were analyzed using fastsimcoal (fig. 2*C*). All five species experienced a drastic rise in effective population size (Ne) ∼10–2.5 Ma (Middle Miocene to Pleistocene), which may reflect ancient range expansion or population growth. Ne decreases from 2.5 to 0.8 Ma to a minimum coinciding with the Naynayxungla glaciation (NG, 0.5–0.78 Ma), which is suggestive of a glaciation-driven bottleneck (Brookfield 1998; Clark et al. 2004; Zhang and Sun 2011). During the Penultimate glaciation (PG, 135–194 Ka), Ne of all three species from clade2 declined from a maximum, whereas the Ne of *T. stewarti* showed a population expansion from a minimum. *T. tibetana* showed a consistently low Ne. After this period, the Ne of all lineages declined until the Last Glacial Maximum (LGM, 26.5–19.0 Ka) except *T. stewarti*. Combined with fastsimcoal results, the increase of Ne for both clade2 species (0.2–1.0 Ma) and *T. stewartia* (26.5–250 Ka) after the NG correspond to their estimated timing of gene flow with *T. tibetana*.

### Window-Based Phylogenomic Analysis of Introgressed Regions

**Fig. 3.**
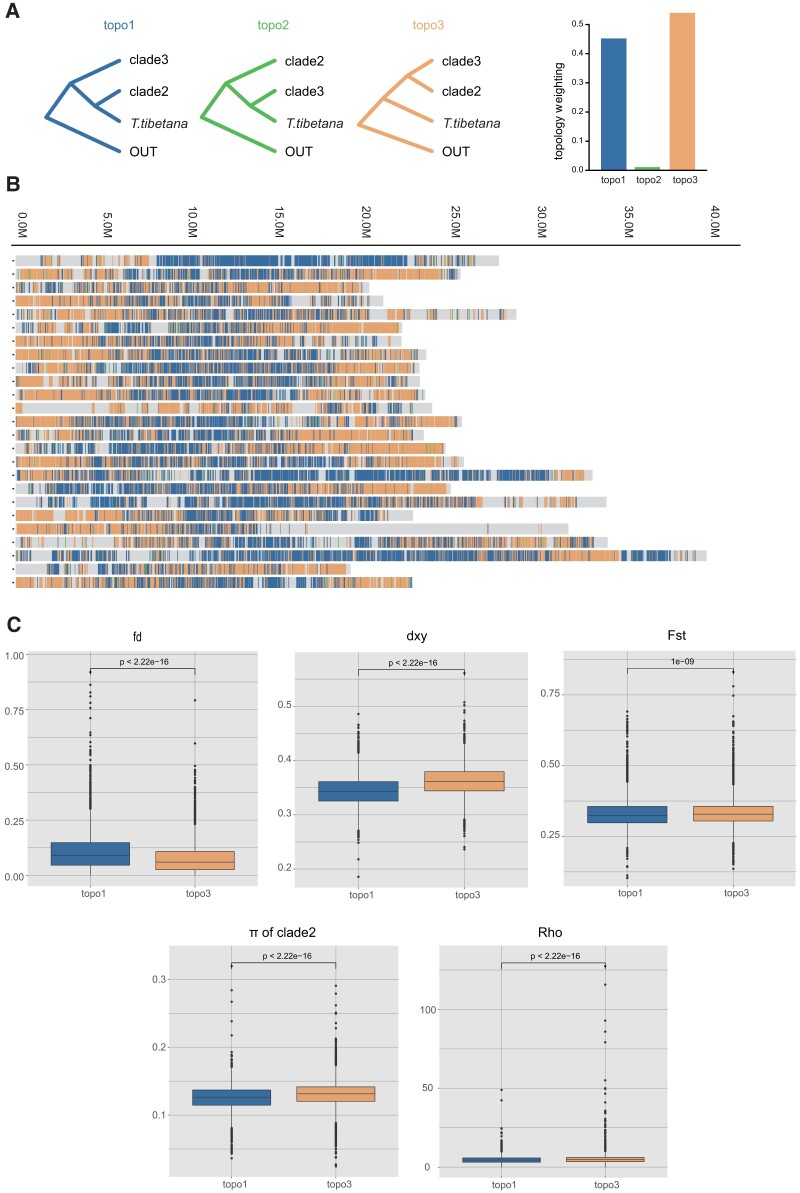
Window-based population genomic analysis. (*A*) Three possible phylogenetic topologies inferred from 25-kb window local ML trees for *T. tibetana* clade2 and clade3, with *P. dabryanus* as outgroup species. The colored vertical bars represent the windows with complete monophyly of the three alternative topologies, where the proportion of the three topologies is the average value of three cade2 species. (*B*) Distribution of tree topologies across the genome. Colors are consistent with the topology in *A*. (*C*) Population genomic parameters of two different topological regions between *T. tibetana* and clade2.

We further analyzed the strong admixture signals between *T. tibetana* and clade2 indicated from population structure analysis, the introgression analysis, and the evidence of mtDNA introgression from mitonuclear discordance in *Triplophysa* phylogeny (Wang et al. 2016). We used the 25k window-based topology weighting analysis between *T. tibetana* and clade2 to define the topology type of each window, with clade3 as the third species and *P. dabryanus* as outgroup. We calculated the proportions of support for topo1, topo2, and topo3, at 45.7%, 1.0%, and 53.3% in all 25k window local trees, respectively (fig. 3*A*). The distribution of windows supports topo1 and topo3 across whole genomic regions (fig. 3*B*). These two tree topologies were supported by 32.3% and 37.7% of the genome, respectively, with a distribution characteristic of topo1 concentrating to the middle of the chromosome and topo3 to both ends in most chromosomes.

To confirm the relationship between introgressed loci and these two topological regions, we used a *f_d_* statistic to calculate the fraction of introgression from *T. tibetana* to clade2 in 25k nonoverlap sliding windows (Martin et al. 2015). The results showed that the topo1 regions had significantly higher *f_d_* values, indicating that *T. tibetana* introgression into clade2 occurred predominantly in these regions, consistent with the hypothesis that *T. tibetana* is sister to clades2 indicative of introgression between them. To evaluate whether there is an association between natural selection and the distribution of different topological regions, we examined a number of population genetic factors that would be impacted by natural selection. The 25k window-based population parameters, including Fst, absolute genetic divergence (Dxy), and nucleotide diversity (*π*), between *T. tibetana* and clade2, were significantly higher in windows supporting topo3 than those in supporting topo1, and the population recombination rate also presented a consistent trend of significantly lower value in topo1 (Wilcoxon rank sum test; fig. 3*C*). In general, introgression in topo1 is mainly concentrated in genomic regions with lower Dxy, Fst, nucleotide diversity, and recombination.

## Discussion

In this study, we used data sets from genome-wide variants to reconstruct phylogenetic trees and histories of discordance in high-altitude loaches. Despite the differences across different types of sequence data sets and analyses, most of the phylogenetic relationships inferred are generally consistent. There are four major clades comprising the 18 *Triplophysa* species included in our study, and we uncovered evidence for interspecies gene flow that occurred both in ancient as well as recent times and is consistently supported as the source of phylogenetic inconsistency.

Different from previous studies (Wang et al. 2016; Wang, Zhang et al. 2020), our phylogenetic results indicate genetic exchange among *Triplophysa* species in geographically adjacent regions that probably resulted from hybridization among microallopatric lineages (Toews and Brelsford 2012; Wang et al. 2018). Mitonuclear discordance can be an evidence of historical interspecies hybridization and mitochondrial capture between ancestors of the *T. tibetana* and *T. stewarti* lineages, consistent with fastsimcoal analyses, which were detected. However, the mitochondrial phylogeny did not reveal hybridization events between *T. tibetana* and clade2, possibly due to alternative factors such as local adaptation of mtDNA genes (Pavlova et al. 2013), asymmetric hybridization (Chan and Levin 2005), or mitochondrial introgression or fusion following secondary contact (Phuong et al. 2017; Garrick et al. 2019).

Generally, individual gene trees may differ from the species phylogeny due to gene duplication, ILS, or introgressive hybridization (Avise and Robinson 2008; Degnan and Rosenberg 2009). Phylogenetic analyses based on concatenated data do not take into account how the stochasticity of the coalescent process affects the accuracy of an obtained species tree and this bias does not diminish as the amount of data increases (Kubatko and Degnan 2007). We found another factor that contributes to inconsistency in phylogenetic construction: The source of the variation in sites used to infer phylogenetic relationships, if they are due to hybridization. In this study, the topology with the highest proportion in topology weighting analysis was different when infer from local window tree sets and gene tree sets (supplementary fig. S6, Supplementary Material online), so did the topology of ASTRAL species tree constructed from those two sets.

When using the gene trees, CDS region and 4D sites, the inferred phylogenetic relationships of *T. tibetana* and four major clades are always consistent (supplementary fig. S5*g*–*i*, Supplementary Material online). This kind of pattern was first found in a study of the radiation of *Lonchura* munia birds, in which trees inferred from autosomal regions thought to relate to color evolution differed significantly from tree inferences based on genome-wide SNPs (Stryjewski and Sorenson 2017). These results are consistent with the hypothesis that sampling smaller sets of genomic loci may help avoid the effects of gene flow due to stochastic sampling during phylogenetic inference, in the premise of most genomic loci misrepresent the real phylogeny (Alstrom et al. 2018; Zhang et al. 2021).

The success of introgressed adaptive traits depends on the rates of hybridization, introgression, recombination, and the strength of selection (Lenormand 2002; Ghalambor et al. 2003). A high rate of hybridization with little opportunity for recombination and/or selection may not necessarily lead to the evolution of adaptive traits. In contrast, a low rate of hybridization with an opportunity for recombination and selection will more likely lead to the incorporation of adaptive traits and simultaneous purging of maladaptive traits within progressively shorter time intervals (Feulner et al. 2013; Arnold and Kunte 2017). Our results suggested that signatures of introgression were preserved between *T. tibetana* and clade2 due to lower rates of recombination and the lower Dxy, Fst, and nucleotide diversity may be a signal of selection across this pair.

One potential explanation for the strong signals of introgression between *T. tibetana* and clade2 found in this study is that ancient admixture of ancestral variation occurred between *T. tibetana* and ancestral lineage of clade2. Previous studies have shown that genes associated with energy metabolism and hypoxic responses have undergone positive selection and rapid radiation in *Triplophysa* fishes (Wang et al. 2015). Positive selection frees the hybridized alleles from the fate of being lost due to genetic drift (Zhang et al. 2021). However, intrinsic factors alone are insufficient to explain bursts of diversification and adaptive radiation (Pease et al. 2016). Our reconstruction of the demographic history of *Triplophysa* suggests that this clade underwent an explosive speciation event corresponding to the second episode of Tibetan Plateau uplift (supplementary fig. S7, Supplementary Material online). At the late stage of the second uplift from 10 to 4 Ma, climate change caused by the monsoon cycle led to rapid major global cooling (Shi and Li 1999). Concurrently, the Ne of *Triplophysa* expanded stably during this period (fig. 2*C*). Due to the lack of strict reproductive isolation between early differentiated species, gene flow between ancestral lineages may also have occurred during this time (Marques et al. 2019). During the third episode of Tibetan Plateau uplift from 4 Ma to the present, the Ne of *Triplophysa* gradually decreased and presented distinct population histories in the subsequent three glaciation events.

It is thought that cold-adapted species undergo range expansions southward and into lowlands during glaciations (Sanchez-Montes et al. 2019; Dufresnes et al. 2020). After the NG ice age retreat, all species except *T. tibetana* tended to return to higher effective population size. This may be related to the extensive gene flow between *Triplophysa*. In our results, both inferred gene flows temporally coincide with an increase in the effective population size of *T. stewarti* and clade2. Thus, episodes of introgression that affect adaptive loci can promote species persistence in regions experiencing high climatic turnover through time.

However, adaptive introgression is only one potential consequence of gene flow; admixture may also result in maladaptively admixed individuals. Deleterious alleles are expected to be removed from a population by random genetic drift alone in a population of small Ne, and gene flow that results in masking of deleterious alleles is the key to sustain the growth rate of populations (Whiteley et al. 2015; Hoffmann et al. 2021; Khan et al. 2021). Stratigraphic studies show that the Tibetan Plateau has undergone three uplifting events since the late Cenozoic and that these have had a profound impact on the East Asian environment and by implication on the biodiversity of the region (Shi and Li 1999; Zhisheng et al. 2001; Li et al. 2015). A violent and uneven tectonic uplift called Gonghe Movement that occurred about 150 thousand years ago divided the river into independent closed lakes or cut off the connection between the water systems (Shi and Li 1999; Yu et al. 2022). This change may have also geographically isolated some *Triplophysa* species in the region, and it is very likely that the *T. tibetana* formed a small, isolated population during this period, as suggested by both PSMC and fastsimcoal simulations (fig. 2*B* and *C*). K-mer-based analysis showed that the *T. tibetana* has a low levels of heterozygosity (Yang, Liu, et al. 2019), and as other species that have gone through recent population size declines, *T. tibetana* also shows low genetic diversity (supplementary fig. S1, Supplementary Material online). This might be due to a possible founder effect for the *T. tibetana* population (Xue et al. 2015; Westbury et al. 2018; Liu et al. 2021).

In general, our study confirms that introgression poses certain challenges in clarifying the evolutionary relationships between *T. tibetana* species. Our work confirms the link between this introgression and selection, which is important for exploring the molecular genetic mechanisms behind the adaptive evolution of *T. tibetana*. At the same time, it is of great significance for genetic communication between species, maintenance of species diversity, and adaptive evolution. Our investigation of *T. tibetana* population history is useful for understanding changes in population dynamics. At a time when the habitat of the *Triplophysa* is increasingly fragmented, these endangered populations are in urgent need of conservation.

## Materials and Methods

### Sample Collection, Whole-Genome Resequencing and Genotype Calling

A total of 138 samples were collected for this study, representing 18 species of the *Triplophysa* genus. An individual of *P. dabryanus* was used as an outgroup (supplementary table S1, Supplementary Material online). Total genomic DNA was extracted from the muscle tissue, and 150-bp PE libraries were sequenced on an Illumina HiSeq X Ten platform. Before reads mapping, we used fastp (version 0.20.0) to remove adapter sequences and to trim low-quality bases with default parameters. Sequence reads were aligned to the *T. tibetana* reference genome (https://www.ncbi.nlm.nih.gov/assembly/GCA_008369825.1/) (Yang, Liu, et al. 2019) using bwa-mem (version 0.7.15) with default parameters. SNVs and short-indel variants were called using the GATK (version 4.1.0.0) with the filter expression “QUAL < 30.0 || QD < 2.0 || FS > 60.0 || MQ < 40.0 || SOR > 4.0” to discard site types probably caused by mapping bias and following cutoffs: minimum depth > 20, minor allele frequency > 0.05, missing rate < 0.5, and only keep biallelic sites. Finally, a total of 11,683,331 sites that passed all these filtering criteria were used in further phylogenetic analyses.

### Phylogenetic Relationships Analysis

To infer the phylogenetic relationship of the 18 species, we used concatenation approach and multispecies coalescent approach (Degnan and Rosenberg 2009). We inferred a ML tree based on three data sets by concatenating all filtered SNPs across the WGS, protein CDS, and 4D sites, respectively. A total of 461,486 4D sites were extracted. For the multispecies coalescent approach, we first split the whole genome into non-overlapping windows of 200, 100, 50, 25, and 10 kb. We reconstructed the ML trees for each window with <100 sites filtered out. We extracted SNPs from gene regions and kept one sample with the highest coverage in each species. From 24,138 annotated genes in a *T. tibetana* reference genome, a total of 3,600 genes were used to reconstruct gene trees in IQTree with the same manner as for our sliding window local tree analysis and a filter of >500 SNP loci. ASTRAL species trees were then inferred from the six genomic windows/gene tree data sets (Mirarab et al. 2014). All ML trees were inferred using IQTree with a GTR + ASC substitution model and 1,000 bootstrap replicates. We plotted a subset of 1,000 randomly selected 200-kb window ML trees in the program DensiTree (Bouckaert et al. 2019). The topology weighting analysis between each phylogenetic clade and *T. tibetana* species, which shows different sizes of sliding windows supporting all possible topologies, was conducted from sliding window-based local ML trees using Twisst (Martin and Van Belleghem 2017).

### Population Genetic Structure Analysis

We used a linkage disequilibrium filter (Zhou et al. 2020) implemented in PLINK (v1.90b6.13 64-bit) (Purcell et al. 2007) with the parameter “indep-pairwise 100 kb 1 0.8” to generate a data set of 1,746,277 SNPs for the population genetic analysis. PCA was performed with the Genome-wide Complex Trait Analysis (GCTA, version: 1.25.3) (Yang et al. 2011). We used the software ADMIXTURE (Alexander et al. 2009) with a random seed to infer population structure of 18 species. The number of genetic clusters (*K*) was set from 2 to 20. We estimated nucleotide diversity (*π*) among 18 species of *Triplophysa* using VCFtools (0.1.15).

### Analyses of Introgression

The outgroup *f3*-statistic and *D*-statistics were calculated using the qp3pop and qpDstat programs in ADMIXTOOLS (Patterson et al. 2012) to detect gene flow between all *Triplophysa* fishes. Outgroup *f3* statistics were performed in the form of (PDAB; A and B), where A and B represent a pair of species of *Triplophysa* and PDAB represents outgroup *P. dabryanus*. The expected value was proportional to the shared genetic history between A and B: The larger the *f3* value, the greater the genetic relatedness between the two populations. For the *D*-statistics, we used the quartet topology form (W, X, Y, and OUT), where W, X, and Y represent populations from clade1, clade2, clade3, clade4, and the TTIB population in a species tree. All 816 detected quartets conformed to the species tree. We considered a *Z*-score of >3 and a positive value of *D*-value indicative that a W population shared genetic admixture with a Y population in a test form and identified a species pair W_Y. A *D*-value less than −3 indicated that an X population shared genetic admixture with a Y population in a test form and identified as a species pair X_Y. All *D*-statistics were classified as 136 species pairs.

We then employed QuIBL (Edelman et al. 2019), a method based on internal branch length distribution, to quantify the proportions of ILS and introgression contributions to topology incongruence. As a tree-based method, the results of QuIBL are trusted to the assumption that input trees are inferred from loci with limited internal recombination. We therefore chose the gene tree set for analysis. We used the species tree topology to assign the outgroup to each triplet in QuIBL. For each triplet form W_X_Y with outgroup Y, we also identified a species pair as W_X. After filtering with the significance determination value |dBIC| > 10, we found 132 discordant species pairs and calculated the average of the total IntroProp for each species pair.

### Demographic History Reconstruction

We used PSMC (Li and Durbin 2011) to estimated effective population size changes through time based on whole-genome variants of six individuals: TTIB.YLD0088, TSTE.YLD0087, TSHI.C10, TSTR.D7, and TSTO.A10, representing *T. tibetana*, *T. stewarti*, *T. shiyangensis*, *T. strauchii*, *T. stoliczkai*, respectively. The consensus sequences were generated using vcfutils.pl (vcf2fq -d 10 -D 80). The fq2psmcfa tool was used to create the input file for PSMC modeling. Sequences were used as the input for the PSMC estimates with the options -N25 -t15 -r5.

Given the high gene introgression levels exhibited in *D*-statistic analyses between *T. tibetana* and clade2, as well as exhibited between *T. tibetana* and *T. stewarti*, we used a coalescent simulation-based method employed in fastsimcoal2.7 (Excoffier et al. 2013) to infer the demographic histories for those three population. We selected two samples from each species in clade2 (*T. shiyangensis*, *T. strauchii*, and *T. stoliczkai*) and joined them together as a whole population named clade2. Populations of *T. tibetana* and *T. stewarti* contained three and six individuals, respectively. We used the two-dimensional joint SFS (2D site frequency spectrum) constructed from sample allele frequencies using angsd (v0.934) (Nielsen et al. 2012). To minimize the bias in demographic inferences due to selection, only neutral sites (4D) were used for this analysis. A total of 325,487 segregating sites were filtered from all 473,182 4D sites. The setting of the model is mainly based on the assumption that introgression and migration events between occurred three *Triplophysa* groups. The eight models differed in 1) whether gene flow was present between all three species; 2) whether before-divergence gene flow between *T. tibetana* and clade2 was present; and 3) the time, pattern, and migration rate size of gene flow between the three *Triplophysa* species. Three demographic models (supplementary fig. S8*a*–*c*, Supplementary Material online) assumed no gene flow, gene flow between three populations after TDIV0, or gene flow between A0 and *T. tibetana*. In another five demographic models, we add TS as time split point, where one or more gene flows between three populations occurred before or after TS (supplementary fig. S8*d*–*h*, Supplementary Material online). Several parameters were used to define evolutionary scenarios. TDIV1 was used as the divergence time between *T. tibetana* and ancestral lineage of *T. stewarti*/clade2 (named A0), and TDIV0 was used as the divergence time between *T. stewarti* and clade2. The ML estimates for all demographic parameters under each model were obtained from 50 independent runs, with 100,000 simulations applied for parameter estimation (-n 100,000) and 40 loops (-L 40) to perform during likelihood maximization. Model comparison was based on the maximum value of the likelihood using the AIC and Akaike weight. The model with the maximum Akaike weight value was chosen as the optimal one. A generation time of 2 years (Zhou et al. 2021) and a mutation rate of 4.13e−9 (Fu et al. 2010) per million years were applied in simulations.

### Divergence Time Estimation

We estimated the lineage divergence times based on 25-kb window data set. Only SNVs in the windows concordant with the consensus phylogeny (5.13 million SNVs) were used (Yang et al. 2021). We then used MCMCtree from the package PAML (Yang 2007) to estimate divergence times with the independent rate model (clock = 2). The MCMC run was executed with 2,000 generations as burn-in and sampled every 10 generations until a total of 20,000 samples were collected.

### Window-Based Genealogy Difference Analysis and Population Genomic Analysis

We used Twisst to assess and quantify the phylogenetic discordance among *T. tibetana* (TTIB), clade3, and clade2 along the genome. The genealogical relationships of these species can be defined by three possible topologies: ((TTIB, clade2), clade3); ((TTIB, clade3), clade2); ((clade2, clade3), TTIB). *Paramisgurnus dabryanus* was used as an outgroup species, and local phylogenetic subtrees were inferred from 25-kb window local ML trees. The genealogy type with the highest percentage in topology weightings was determined as the type of the window. We estimated window-based population parameters including Fst, absolute genetic divergence (Dxy), nucleotide diversity (*π*) using the genomics_general package with 25-kb nonoverlapping sliding windows (https://github.com/simonhmartin/genomics_general). The package is also used to calculate *f_d_* and estimate the proportion of introgression in the same window (Martin et al. 2015). Under a given four-taxon topology ((clade3, clade2), TTIB, OUT), a positive *f_d_* statistic value indicates the introgression proportion from population TTIB to clade2. The minimum sites used in each window were set to 3 through -m flag. For windows of *D* < 0, or of *D* > 0, but *f_d_* > 1, the *f_d_* statistic value becomes meaningless, and we filtered it out. We estimated the population recombination rate (Rho) for species of clade2 in FastEPRR 2.0 (Gao et al. 2016) in the same window size.

## Supplementary Material

msad129_Supplementary_Data

## Data Availability

Illumina whole genome resequencing reads for 18 species of *Triplophysa* are available at Genome Sequence Archive (GSA https://ngdc.cncb.ac.cn/gsa/) under BioProject PRJCA015528. Results of D-statistic and QuIBL analyses are available as Supplementary material online. All templates used in the fastsimcoal analysis are available on GitHub at https://github.com/rainstop2019/Project_2022. ***Conflict of interest statement*.** The authors declare no competing interests.
