# Supplementary material for: The Role of Introgression During the Radiation of Endemic Fishes Adapted to Living at Extreme Altitudes in the Tibetan Plateau
Source: Mol Biol Evol. 2023 May 29;40(6):msad129. doi: 10.1093/molbev/msad129 (PMC10297026; doi:10.1093/molbev/msad129)
Supplement: msad129_Supplementary_Data [file msad129_supplementary_data.zip › Supplementary Material.pdf]

Table of contents:

**Figure number**	**Page**
Figure S1 Boxplot of nucleotide diversity (π)	1
Figure S2 Structure analysis with Admixture	2
Figure S3 Structure cross validation error plot	3
Figure S3 Pairwise out-group f3 test heat map	4
Figure S5 Summary of topologies among multi-method and multi-dataset	5-6
Figure S6 Alternative phylogenetic topologies statistics with twisst	7
Figure S7 Time-calibrated tree of *Triplophysa* genus estimated by MCMCTree	8
Figure S8 Eight tested demographic models for the speciation and migration process of *Triplophysa*	9
Table S1 Sequencing and mapping summary for all samples included in the study	10-13
Table S2 Sample Information	13
Table S3 Methods for phylogenetic framework	14
Table S4 ASTRAL quartet score statistic	14
Table S5 Comparison of demographic models analyzed with FASTSIMCOAL2.	14
Table S6 Gene Ontology term enrichment in topo1 type genomic region	15-16
Table S7 KEGG pathways enrichment in topo1 type genomic region	17-18

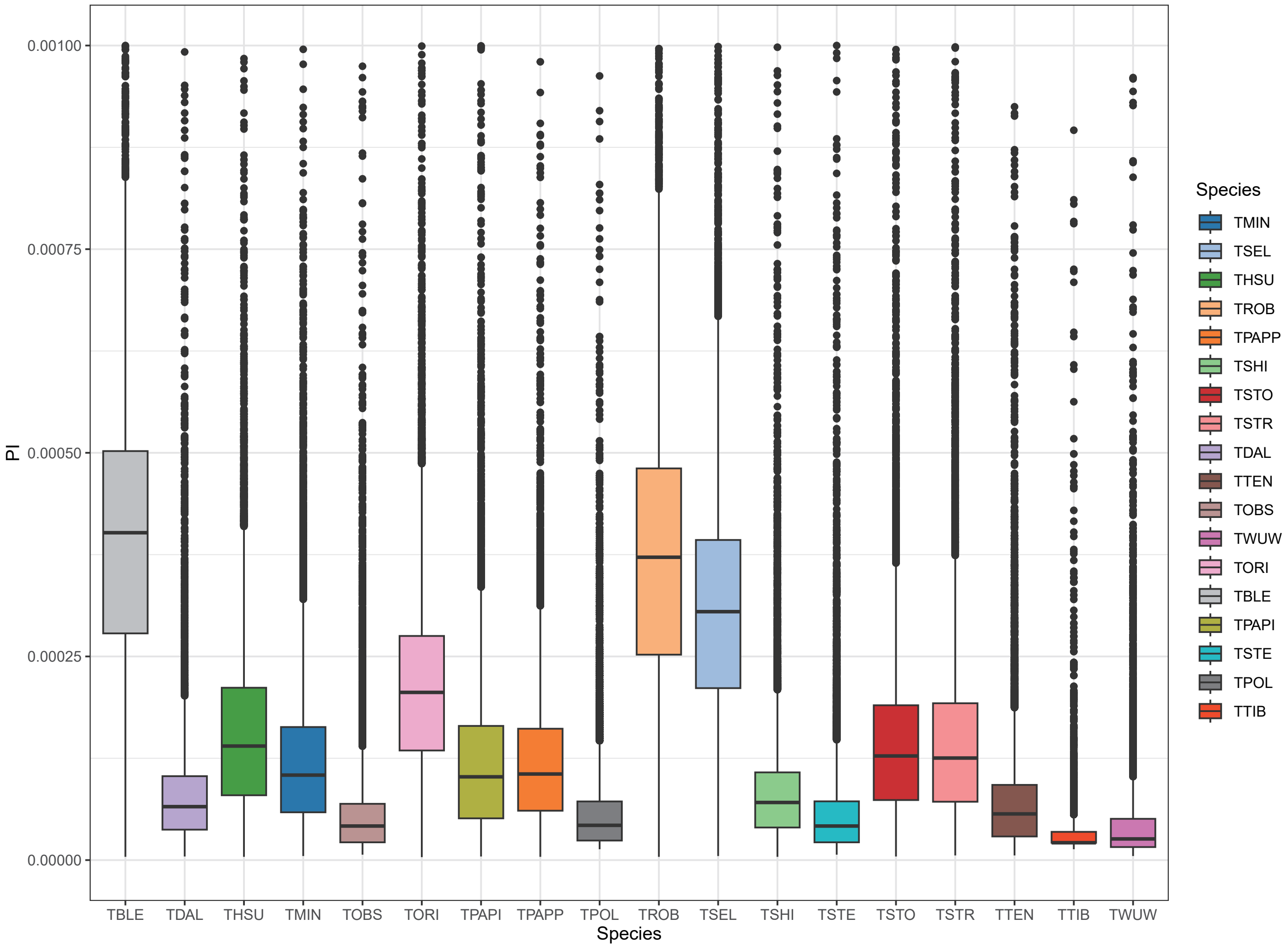

Figure S1. Boxplot of nucleotide diversity (π) for 18 *Triplophysa* species.

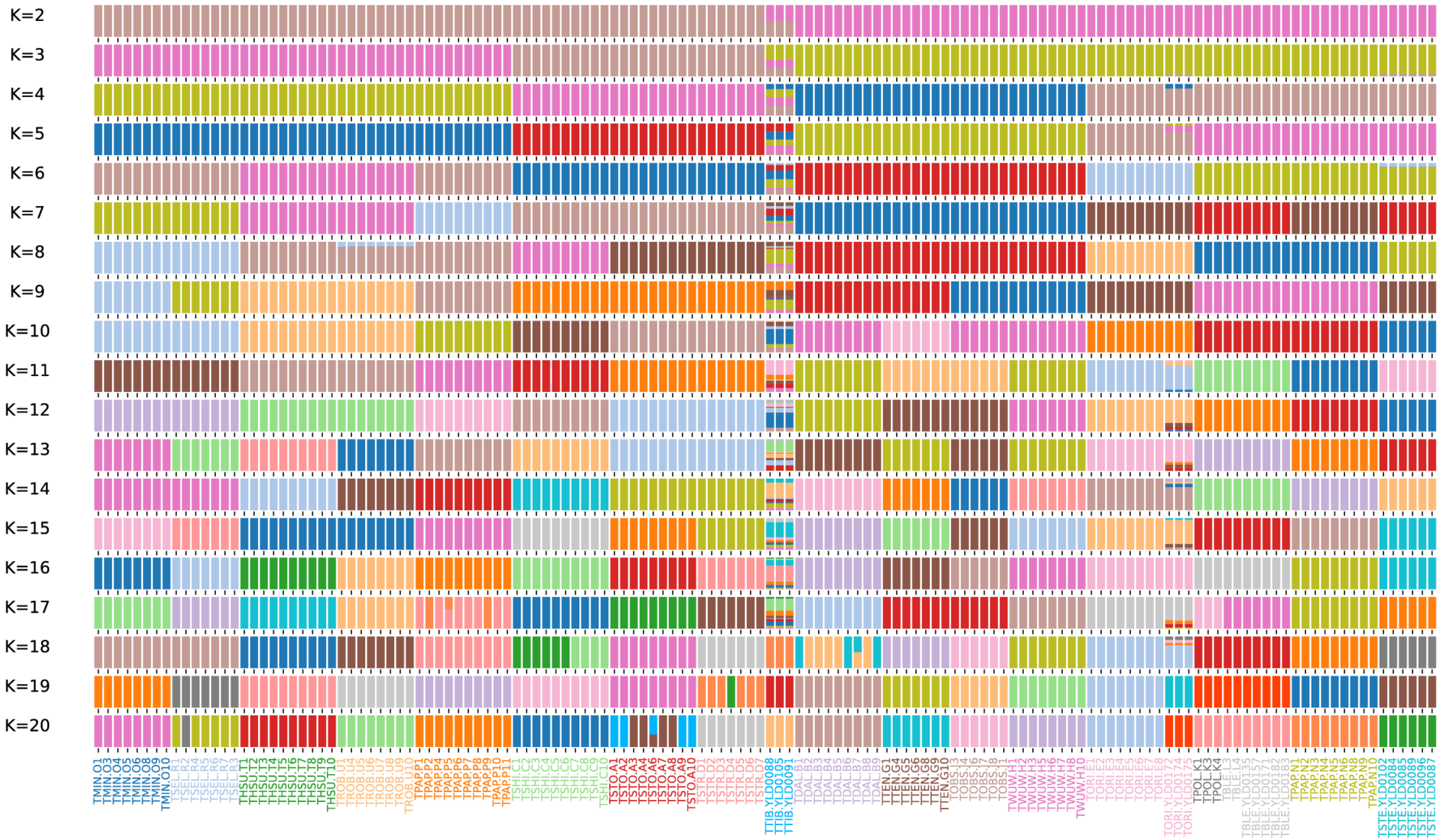

Figure S2. Summary plot for *Triplophysa*. Each individual is represented by a single vertical line broken into K colored segments with lengths proportional to each of the K inferred clusters, where the K values are shown in the legend. The individual IDs are presented at the bottom of the plot.

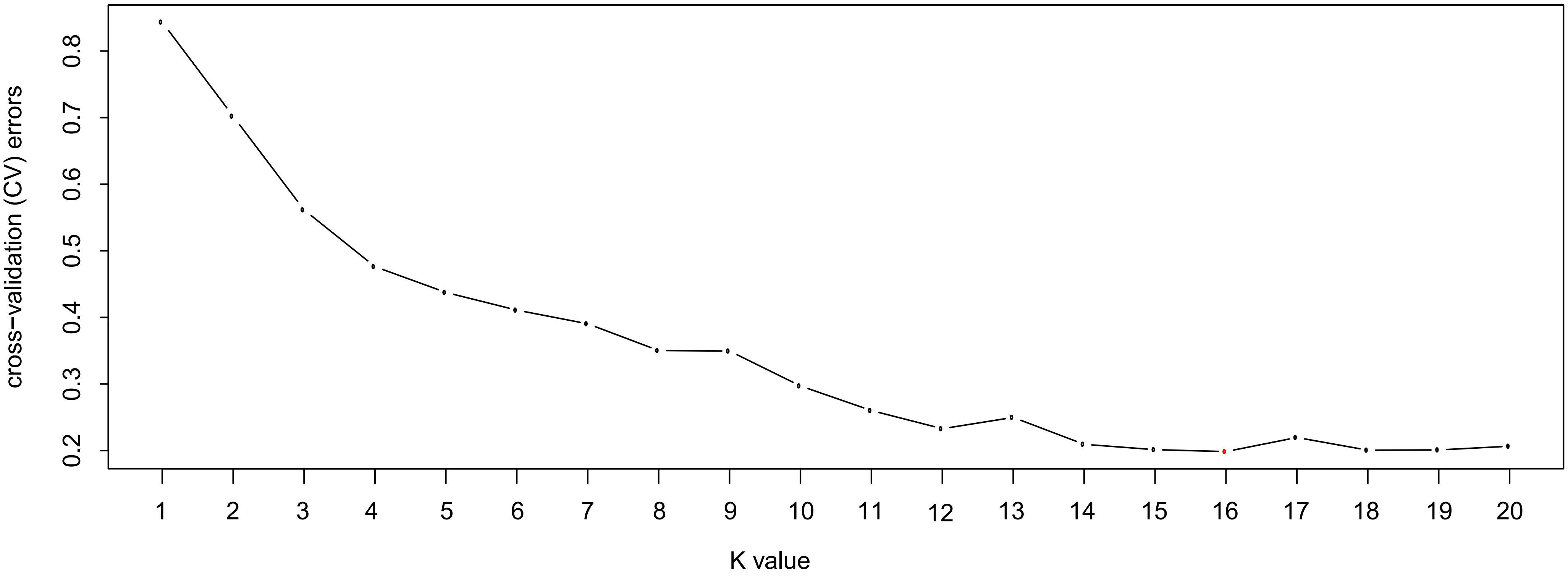

Figure S3. The cross validation error estimated by ADMIXTURE for different model K. The red dot represents the best K value K=16.

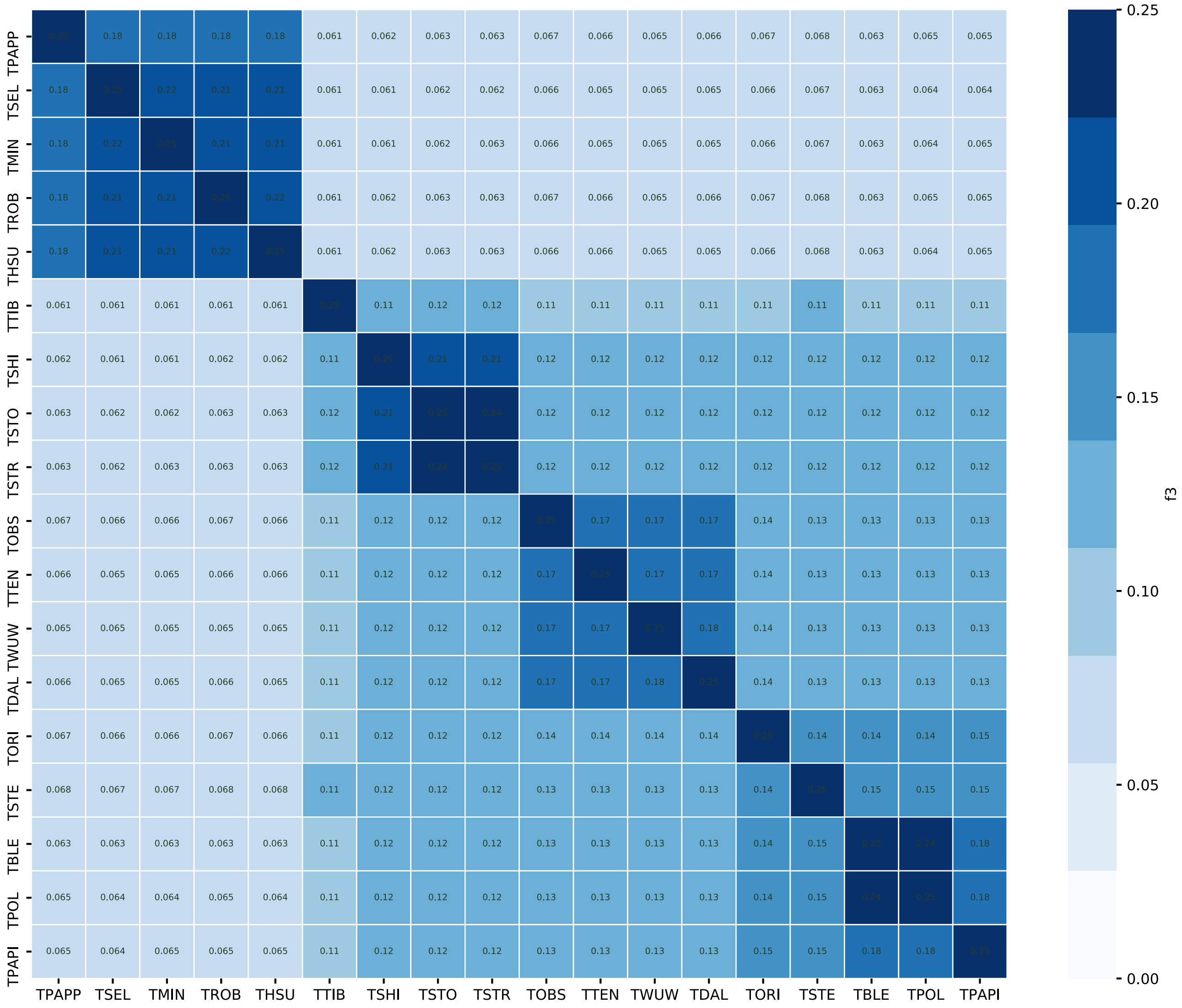

Figgure S4. Pairwise out-group *f3* estimate value heatmap among *Triplophysa* populations in the form *f3*(P.dabryanus; X ,Y). All tests get a significant result for |Zscore| ≥ 3.

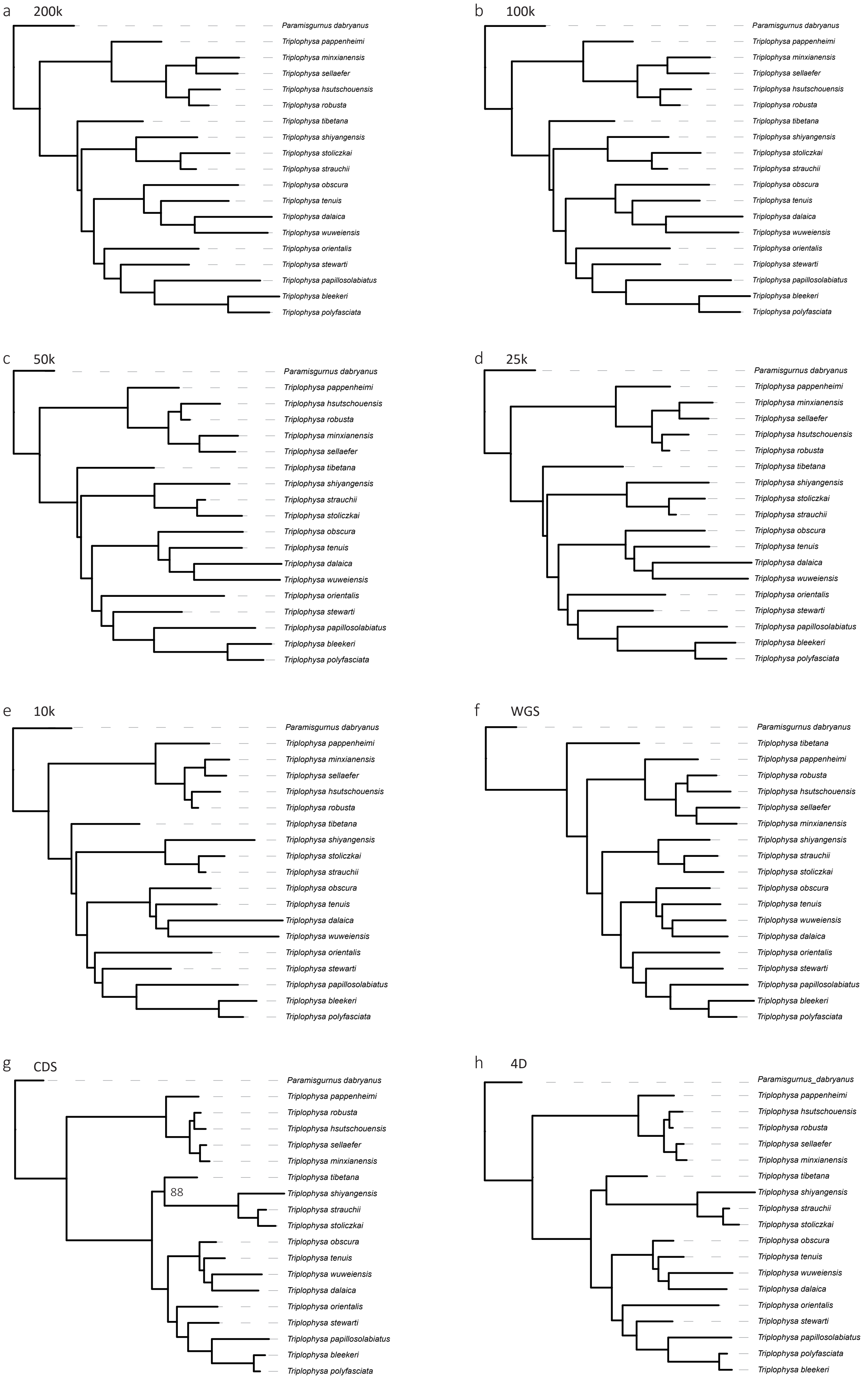

a 200k
Paramisgurnus dabryanus
Triplophysa pappenheimi
Triplophysa minxianensis
Triplophysa sellaefer
Triplophysa hsutschouensis
Triplophysa robusta
Triplophysa tibetana
Triplophysa shiyangensis
Triplophysa stoliczkai
Triplophysa strauchii
Triplophysa obscura
Triplophysa tenuis
Triplophysa dalaica
Triplophysa wuweiensis
Triplophysa orientalis
Triplophysa stewarti
Triplophysa papillosolabiatus
Triplophysa bleekeri
Triplophysa polyfasciata
b 100k
Paramisgurnus dabryanus
Triplophysa pappenheimi
Triplophysa minxianensis
Triplophysa sellaefer
Triplophysa hsutschouensis
Triplophysa robusta
Triplophysa tibetana
Triplophysa shiyangensis
Triplophysa stoliczkai
Triplophysa strauchii
Triplophysa obscura
Triplophysa tenuis
Triplophysa dalaica
Triplophysa wuweiensis
Triplophysa orientalis
Triplophysa stewarti
Triplophysa papillosolabiatus
Triplophysa bleekeri
Triplophysa polyfasciata
c 50k
Paramisgurnus dabryanus
Triplophysa pappenheimi
Triplophysa hsutschouensis
Triplophysa robusta
Triplophysa minxianensis
Triplophysa sellaefer
Triplophysa tibetana
Triplophysa shiyangensis
Triplophysa strauchii
Triplophysa stoliczkai
Triplophysa obscura
Triplophysa tenuis
Triplophysa dalaica
Triplophysa wuweiensis
Triplophysa orientalis
Triplophysa stewarti
Triplophysa papillosolabiatus
Triplophysa bleekeri
Triplophysa polyfasciata
d 25k
Paramisgurnus dabryanus
Triplophysa pappenheimi
Triplophysa minxianensis
Triplophysa sellaefer
Triplophysa hsutschouensis
Triplophysa robusta
Triplophysa tibetana
Triplophysa shiyangensis
Triplophysa stoliczkai
Triplophysa strauchii
Triplophysa obscura
Triplophysa tenuis
Triplophysa dalaica
Triplophysa wuweiensis
Triplophysa orientalis
Triplophysa stewarti
Triplophysa papillosolabiatus
Triplophysa bleekeri
Triplophysa polyfasciata
e 10k
Paramisgurnus dabryanus
Triplophysa pappenheimi
Triplophysa minxianensis
Triplophysa sellaefer
Triplophysa hsutschouensis
Triplophysa robusta
Triplophysa tibetana
Triplophysa shiyangensis
Triplophysa stoliczkai
Triplophysa strauchii
Triplophysa obscura
Triplophysa tenuis
Triplophysa dalaica
Triplophysa wuweiensis
Triplophysa orientalis
Triplophysa stewarti
Triplophysa papillosolabiatus
Triplophysa bleekeri
Triplophysa polyfasciata
f WGS
Paramisgurnus dabryanus
Triplophysa tibetana
Triplophysa pappenheimi
Triplophysa robusta
Triplophysa hsutschouensis
Triplophysa sellaefer
Triplophysa minxianensis
Triplophysa shiyangensis
Triplophysa strauchii
Triplophysa stoliczkai
Triplophysa obscura
Triplophysa tenuis
Triplophysa wuweiensis
Triplophysa dalaica
Triplophysa orientalis
Triplophysa stewarti
Triplophysa papillosolabiatus
Triplophysa bleekeri
Triplophysa polyfasciata
g CDS
Paramisgurnus dabryanus
Triplophysa pappenheimi
Triplophysa robusta
Triplophysa hsutschouensis
Triplophysa sellaefer
Triplophysa minxianensis
Triplophysa tibetana
88
Triplophysa shiyangensis
Triplophysa strauchii
Triplophysa stoliczkai
Triplophysa obscura
Triplophysa tenuis
Triplophysa wuweiensis
Triplophysa dalaica
Triplophysa orientalis
Triplophysa stewarti
Triplophysa papillosolabiatus
Triplophysa bleekeri
Triplophysa polyfasciata
h 4D
Paramisgurnus_dabryanus
Triplophysa pappenheimi
Triplophysa hsutschouensis
Triplophysa robusta
Triplophysa sellaefer
Triplophysa minxianensis
Triplophysa tibetana
Triplophysa shiyangensis
Triplophysa strauchii
Triplophysa stoliczkai
Triplophysa obscura
Triplophysa tenuis
Triplophysa wuweiensis
Triplophysa dalaica
Triplophysa orientalis
Triplophysa stewarti
Triplophysa papillosolabiatus
Triplophysa polyfasciata
Triplophysa bleekeri

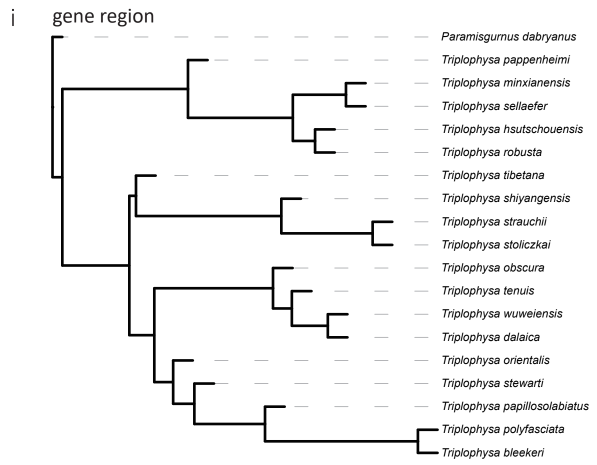

Figure S5. Summary of topologies among multi-method and multi-dataset. **a-e,** The species trees inferred by multi-species coalescent approach (ASTRAL) from fixed-window local trees with different lengths a, 200 kb, b, 100 kb, c, 50 kb, d, 25 kb and e, 10 kb. **f,** Maximum-likelihood (ML) tree of ***Triplophysa*** based on the concatenating SNVs across the whole genome (WGS dataset). **g**, Maximum-likelihood (ML) tree based on only SNVs from sequences of protein-coding genes (CDS dataset). **h**,Maximum-likelihood (ML) tree based on only SNVs concatenating from four-fold degenerate sites (4d-sites dataset). **i**, The species tree inferred by multi-species coalescent approach (ASTRAL) from SNPs concatenating sequences from gene region. Support value (ASTRAL) or bootstrap values(ML tree) are provided next to the nodes unless they were 1 or100.

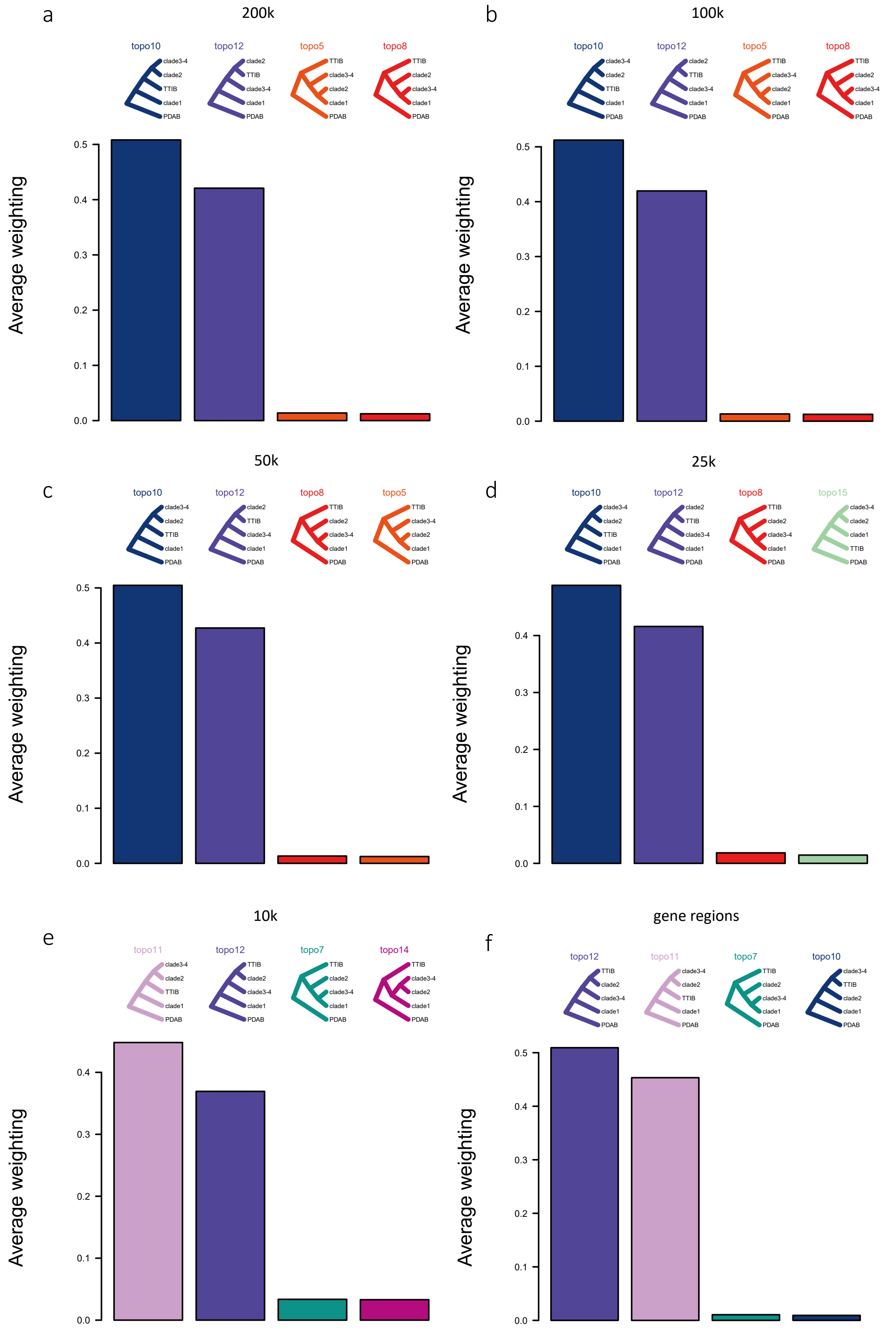

Figure S6. Alternative phylogenetic topologies statistics among clade1,clade2,clade3-4,*T.tibetana* when examining different window-based tree sets and gene tree set, using twisst

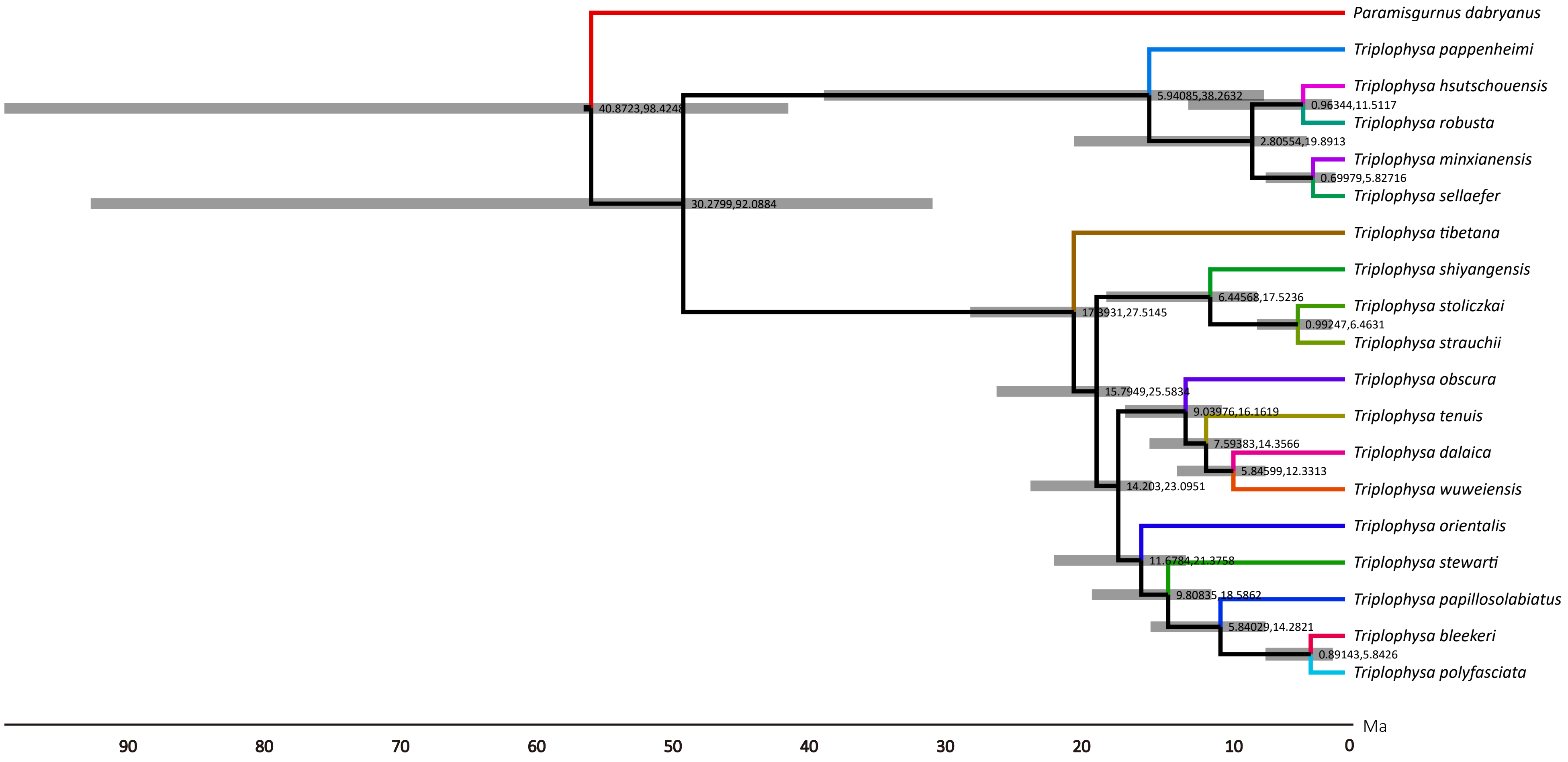

Figure S7. Time-calibrated tree of *Triplophysa* genus estimated by MCMCTree. The gray bars represent the 95% credible intervals of estimated divergence times.

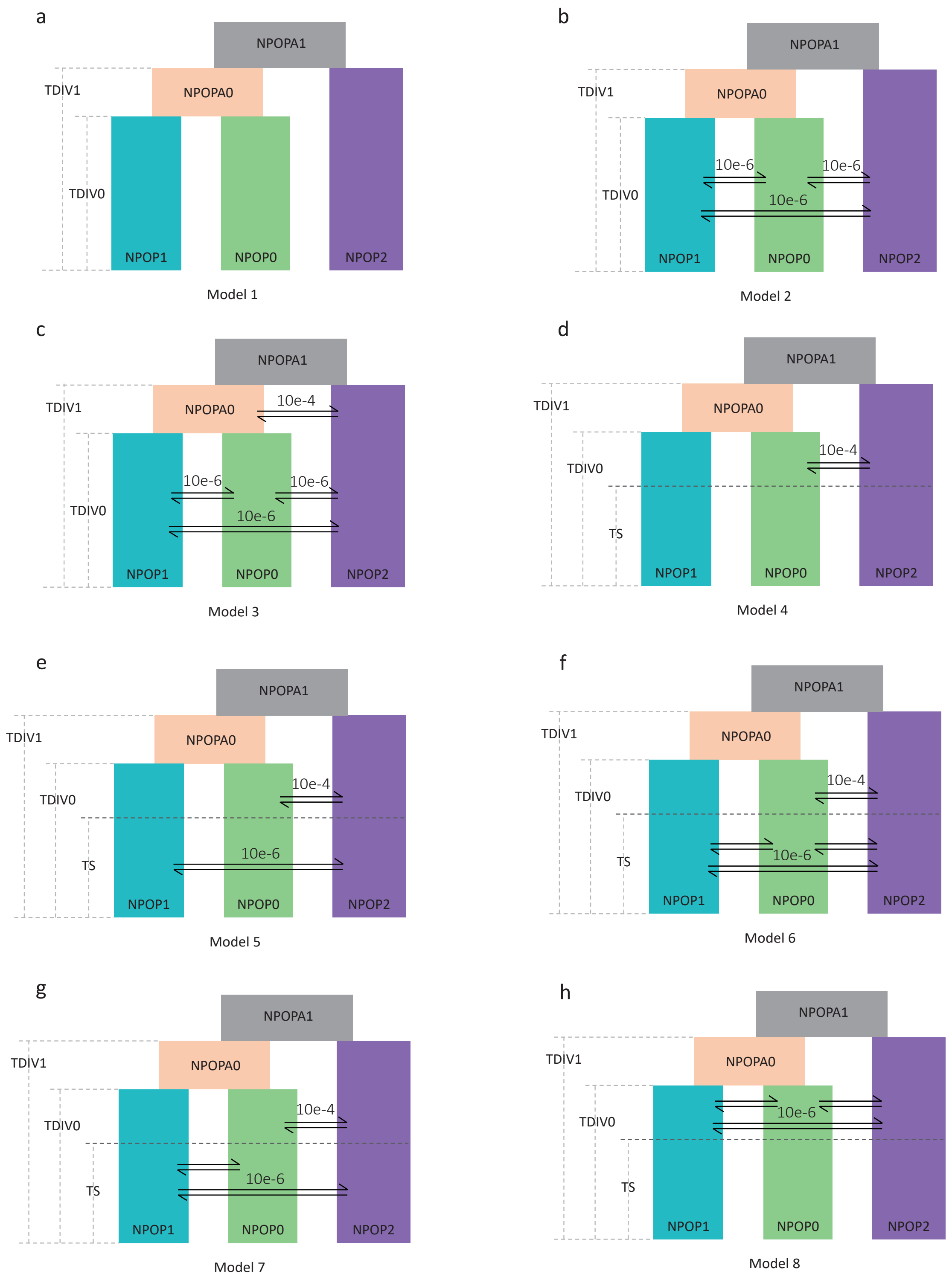

Figure S8. Eight tested demographic models for the speciation and migration process of *Triplophysa*. **a**, among the three populations, no gene flows (Model 1); **b**, gene flow occurred between three populations after TDIV0 (Model 2); **c**, gene flow occurred between three populations, as well as occurred between A0 and *T.tibetana* (Model 3); **d**, gene flow occurred between clade2 and *T.tibetana* after the divergence of *T.stewarti* and clade2 and before recent time TS (Model 4); **e**, similar with d, differed by an additional gene flow occurred between *T.stewarti* and *T.tibetana* after TS (Model 5); **f**, similar with d, differed by three gene flow events occurred between three populations (Model 6); **g**, similar with d, differed by two gene flow events occurred between *T.stewarti* and other two populations (Model 7); **h**, three gene flow events occurred between three populations after the divergence of *T.stewarti* and clade2 and before recent time TS (Model 8).

Table S1. Sequencing and mapping summary for all samples included in the study

Sample ID	TotalReads	MapRate	UniqMapRate	Covrage	Depth	CovrageBase
TWUW.H8	168,134,633	96.52%	74.70%	82.48	27.65	538,541,128
TWUW.H7	145,796,179	96.68%	77.68%	82.21	24.63	536,755,864
TWUW.H6	168,705,806	96.32%	78.19%	82.52	28.28	538,830,144
TWUW.H5	183,291,012	95.42%	78.95%	82.53	30.04	538,857,276
TWUW.H3	137,120,109	96.78%	77.49%	82.21	23.47	536,747,551
**TWUW.H2**	304,485,275	96.64%	76.94%	84.39	51.06	551,024,503
TWUW.H10	178,308,733	96.42%	75.28%	82.6	29.43	539,300,575
TWUW.H1	191,063,477	96.84%	80.27%	82.92	32.07	541,415,670
TTIB.YLD0105	143,008,815	99.51%	92.99%	97.74	31.71	638,200,597
**TTIB.YLD0091**	328,202,916	99.55%	97.22%	98.65	73.85	644,146,070
TTIB.YLD0088	167,207,715	99.55%	95.81%	98.09	37.25	640,484,181
TTEN.G9	142,807,682	97.23%	77.94%	83.89	25.41	547,724,815
TTEN.G8	173,753,936	97.10%	78.95%	84.44	30.89	551,308,529
TTEN.G6	168,851,462	96.89%	79.43%	84.42	29.82	551,224,646
**TTEN.G5**	291,699,217	97.16%	78.16%	85.79	51.78	560,139,509
TTEN.G4	178,181,409	97.19%	79.13%	84.42	31.5	551,221,637
TTEN.G10	147,391,537	91.86%	75.60%	83.95	24.45	548,139,104
TTEN.G1	162,809,406	97.21%	78.98%	84.41	28.65	551,133,122
TSTR.D7	146,093,510	97.64%	78.95%	85.27	26.72	556,779,027
TSTR.D6	196,602,846	97.39%	79.02%	85.95	35.52	561,214,909
TSTR.D5	177,918,574	97.54%	78.90%	85.75	32.06	559,872,219
TSTR.D4	217,332,957	97.68%	77.91%	86.25	39.22	563,128,218
**TSTR.D3**	301,466,089	97.69%	78.32%	86.83	53.82	566,953,172
TSTR.D2	140,093,368	97.75%	78.69%	84.96	25.47	554,745,215
TSTR.D1	145,011,042	97.72%	81.64%	84.8	26.33	553,672,674
TSTO.A9	167,464,222	97.10%	80.53%	84.97	30.42	554,805,973
TSTO.A8	285,014,637	97.47%	80.73%	86.6	51.63	565,415,071
**TSTO.A7**	359,521,383	97.51%	81.23%	86.99	65.41	567,973,233
TSTO.A6	223,269,241	97.59%	80.69%	85.78	40.77	560,069,701
TSTO.A4	201,852,549	97.43%	81.04%	85.61	36.62	558,944,798
TSTO.A3	180,634,436	97.57%	80.83%	84.7	32.92	553,019,529
TSTO.A2	208,092,108	97.63%	80.70%	85.62	37.92	559,020,063
TSTO.A10	209,199,212	97.53%	80.59%	85.8	38.05	560,198,301
TSTO.A1	208,036,879	97.54%	80.67%	85.51	37.91	558,341,294
**TSTE.YLD0102**	353,890,428	97.79%	79.02%	86.46	64.25	564,501,653
TSTE.YLD0096	159,045,778	97.70%	77.71%	84.68	28.78	552,890,292
TSTE.YLD0089	178,006,292	97.79%	79.81%	84.8	32.68	553,672,541
TSTE.YLD0087	311,122,421	97.67%	78.60%	86.36	56.31	563,853,715
TSTE.YLD0085	287,344,131	97.76%	78.88%	86.05	52.2	561,853,552
TSTE.YLD0084	200,922,886	97.83%	79.80%	85.23	36.74	556,505,359
TSHI.C9	207,197,710	97.04%	79.22%	84.87	37.06	554,157,774

TSHI.C8	189,233,209	97.22%	79.30%	84.47	33.77	551,500,940
**TSHI.C7**	300,054,452	97.32%	79.43%	86.02	53.67	561,677,175
TSHI.C6	240,921,970	96.80%	79.17%	85.24	42.86	556,584,912
TSHI.C5	190,493,254	97.41%	79.90%	84.44	34.18	551,306,188
TSHI.C4	178,731,411	97.40%	79.96%	84.37	31.99	550,845,002
TSHI.C3	182,433,788	97.18%	79.51%	84.61	32.73	552,428,632
TSHI.C2	202,000,093	97.38%	80.01%	84.55	36.25	552,022,336
TSHI.C10	169,637,053	97.08%	79.87%	84.21	30.42	549,843,972
TSHI.C1	229,276,878	97.33%	79.48%	85.13	41.07	555,835,479
TSEL.R7	159,317,827	93.84%	70.82%	72.91	24.11	476,058,960
TSEL.R6	241,064,776	93.84%	69.10%	73.84	36.14	482,115,643
TSEL.R5	200,551,772	93.53%	68.21%	73.34	30.03	478,826,975
TSEL.R4	188,224,737	93.43%	68.88%	73.56	28.22	480,296,450
**TSEL.R3**	354,178,747	93.75%	69.29%	75.56	53.44	493,373,233
TSEL.R2	205,881,963	93.85%	68.76%	74.12	30.95	483,955,427
TSEL.R1	181,939,180	93.78%	69.03%	73.57	27.44	480,328,819
TROB.U9	237,561,760	94.13%	70.78%	75.06	36.1	490,110,467
TROB.U8	201,010,414	94.09%	70.14%	74.2	30.46	484,475,402
TROB.U7	157,044,316	93.96%	70.13%	73.56	23.84	480,314,752
TROB.U6	196,239,223	93.72%	68.04%	74.31	29.36	485,163,692
TROB.U5	210,874,972	93.74%	68.15%	74.63	31.39	487,312,966
TROB.U4	205,356,948	94.16%	70.88%	74.11	30.97	483,877,733
TROB.U10	285,974,218	93.62%	69.84%	75.59	42.8	493,576,817
**TROB.U1**	248,165,545	93.67%	69.33%	75.7	37.09	494,240,441
TPOL.K4	146,849,308	97.32%	77.34%	82.08	25.7	535,954,597
**TPOL.K3**	301,235,710	97.16%	76.71%	84.26	52.18	550,152,987
TPOL.K1	154,347,243	97.26%	77.83%	82.25	27.03	537,002,720
TPAP.P9	158,722,743	93.99%	70.83%	73.48	23.83	479,746,705
TPAP.P8	159,559,792	94.11%	70.47%	73.21	23.7	478,004,189
TPAP.P7	239,681,919	94.46%	71.27%	74.42	35.93	485,922,082
TPAP.P6	153,091,558	93.79%	70.37%	73.46	22.8	479,629,388
TPAP.P5	175,718,037	94.22%	69.73%	73.28	26.42	478,457,273
TPAP.P4	169,223,211	93.99%	70.81%	73.39	25.38	479,189,016
TPAP.P2	185,679,735	93.97%	70.38%	73.8	27.84	481,857,143
TPAP.P11	237,747,208	94.16%	71.80%	74.57	35.62	486,892,171
TPAP.P10	135,330,734	94.00%	70.39%	72.85	20.44	475,632,610
**TPAP.P1**	313,021,041	94.10%	70.65%	75.51	46.85	493,002,796
TPAP.N9	183,634,977	97.41%	77.94%	85.18	33.18	556,167,765
**TPAP.N8**	308,998,261	97.21%	76.65%	86.64	54.78	565,687,168
TPAP.N6	291,595,997	97.39%	77.61%	86.2	52.29	562,829,002
TPAP.N5	214,596,892	97.40%	76.62%	85.53	38.16	558,430,826
TPAP.N4	162,411,338	97.38%	78.39%	84.85	29.28	554,011,046
TPAP.N3	180,622,965	97.38%	77.43%	85.15	32.54	555,979,060
TPAP.N2	210,080,489	97.35%	76.30%	85.41	37.11	557,649,436

TPAP.N10	261,281,383	97.58%	77.73%	85.91	46.4	560,958,902
TPAP.N1	146,768,401	97.40%	76.08%	84.6	26.4	552,361,197
TORI.YLD0175	155,443,126	97.11%	81.19%	84.4	28.19	551,083,863
TORI.YLD0174	145,551,748	97.60%	79.39%	84.61	26.56	552,435,900
TORI.YLD0172	142,726,042	97.56%	80.06%	84.62	25.84	552,494,263
TORI.E8	149,422,688	97.61%	77.17%	83.68	26.52	546,340,585
TORI.E7	162,344,113	97.65%	76.96%	84.33	28.71	550,584,979
TORI.E6	220,386,026	97.66%	77.87%	84.64	38.98	552,667,289
TORI.E5	162,771,656	97.59%	77.53%	84.31	29	550,491,229
TORI.E4	157,014,552	97.60%	76.49%	83.82	27.9	547,274,216
TORI.E3	253,068,439	97.56%	78.28%	85.28	44.12	556,803,845
TORI.E2	176,311,056	97.46%	74.79%	84.34	31.01	550,651,119
**TORI.E1**	305,480,685	97.58%	76.19%	85.89	53.62	560,797,628
TOBS.J8	149,195,946	97.31%	77.66%	83.56	26.66	545,601,831
TOBS.J7	162,513,503	97.52%	79.28%	83.24	28.95	543,498,625
TOBS.J6	162,357,868	97.33%	78.70%	83.7	29.1	546,517,631
TOBS.J4	149,982,571	97.47%	78.58%	83.34	26.9	544,151,776
TOBS.J3	139,219,677	97.45%	80.21%	83.47	24.84	544,969,349
**TOBS.J1**	307,504,807	97.40%	77.50%	86.21	54.92	562,874,417
TMIN.O9	181,016,388	93.86%	70.30%	73.64	26.67	480,814,377
TMIN.O8	169,464,447	93.71%	71.01%	73.73	25.02	481,395,767
TMIN.O6	306,796,503	93.38%	69.09%	75.32	45.72	491,818,679
**TMIN.O5**	187,410,393	93.32%	70.08%	78.96	28.09	515,533,579
TMIN.O4	247,296,399	93.22%	69.62%	75	36.58	489,689,335
TMIN.O3	148,659,690	92.91%	68.26%	73.12	21.5	477,412,121
TMIN.O10	227,849,108	93.10%	68.62%	74.47	33.56	486,208,551
TMIN.O1	147,620,686	93.36%	70.06%	73.84	21.9	482,128,112
THSU.T9	149,329,451	93.59%	69.02%	73.98	22.5	483,036,451
THSU.T8	157,931,205	93.72%	68.78%	73.59	23.74	480,515,856
THSU.T7	161,888,517	94.18%	70.75%	73.96	24.62	482,887,162
THSU.T6	189,637,322	93.79%	69.09%	74.19	28.38	484,426,861
THSU.T5	180,987,734	93.30%	69.16%	74.09	26.85	483,741,574
THSU.T4	157,464,047	93.69%	68.54%	72.78	23.25	475,206,822
THSU.T3	177,315,939	93.88%	69.77%	73.08	26.23	477,171,046
THSU.T2	174,471,526	93.60%	69.11%	73.06	25.76	477,053,724
THSU.T10	207,611,336	94.15%	69.86%	74.06	31.24	483,556,118
**THSU.T1**	297,597,987	93.91%	69.21%	75.15	44.26	490,648,431
TDAL.B9	165,879,843	96.58%	79.78%	82.95	29.05	541,635,611
TDAL.B8	162,483,525	90.66%	74.52%	82.67	26.73	539,794,415
TDAL.B7	164,920,233	96.59%	80.15%	82.73	29.02	540,163,829
TDAL.B6	194,174,511	97.09%	80.43%	83.16	34.21	542,955,844
TDAL.B5	200,030,093	96.24%	79.15%	83.41	34.98	544,612,573
**TDAL.B4**	304,751,512	96.77%	79.97%	84.54	53.4	552,005,503
TDAL.B3	205,481,729	97.18%	81.08%	83.05	36.18	542,264,555

TDAL.B2	204,334,758	97.27%	80.93%	83.09	36.03	542,492,328
TDAL.B1	189,655,906	95.80%	78.79%	83.4	33.12	544,566,604
TBLE.YLD0183	149,463,779	96.50%	77.74%	81.71	26.06	533,491,315
TBLE.YLD0182	207,909,291	93.95%	76.74%	82.47	35.38	538,478,919
TBLE.YLD0171	183,279,563	96.83%	79.17%	82.22	32.19	536,853,468
**TBLE.YLD0157**	213,915,110	95.37%	77.51%	82.92	36.82	541,396,217
TBLE.YLD0135	181,255,262	97.03%	78.95%	82.32	31.68	537,467,835
TBLE.L4	281,565,584	97.39%	78.68%	82.52	49.04	538,791,677
TBLE.L3	205,266,050	97.31%	78.60%	81.92	35.76	534,899,124
Average	201,993,163	96.09%	76.21%	81.48	34.27	532,027,938

Table S2 Sample Information

**Sample**	**Species**	**Latitude**	**Longitude**	**Altitude**
TSEL	*Triplophysa sellaefer*	36.21	108.49	1178m
TDAL	*Triplophysa dalaica*	35.17	107.04	1181m
TOBS	*Triplophysa obscura*	39.77	98.62	1403m
TSTR	*Triplophysa strauchii*	39.13	100.39	1412m
TMIN	*Triplophysa minxianensis*	34.80	106.51	1745m
TWUW	*Triplophysa wuweiensis*	38.33	102.02	1827m
TPOL	*Triplophysa polyfasciata*	33.93	106.42	1891m
TTEN	*Triplophysa tenuis*	39.86	96.74	2054m
TSTO	*Triplophysa stoliczkai*	36.31	103.41	2066m
TPAPP	*Triplophysa pappenheimi*	35.47	103.03	2076m
THSU	*Triplophysa hsutschouensis*	37.37	102.92	2273m
TROB	*Triplophysa robusta*	34.40	104.06	2345m
TSHI	*Triplophysa shiyangensis*	38.04	101.95	2465m
TPAPI	*Triplophysa papilloso-labiatus/papillosolabiata*	38.81	98.41	3364m
TORI_1	*Triplophysa orientalis*	34.75	103.01	2928m
TORI_2	*Triplophysa orientalis*	33.57	102.97	3445m
TBLE_1	*Triplophysa bleekeri*	33.57	102.97	3445m
TBLE_2	*Triplophysa bleekeri*	33.44	105.41	1230m
TSTE	*Triplophysa stewarti*	29.63	91.18	3645m
TTIB	*Triplophysa tibetana*	29.63	91.18	3645m

Table S3 Methods for phylogenetic framework

Dataset	sites/trees	Strategy
WGD SNPs	11,683,331 sites	concatenation approach MLtree + IQtree
CDS SNPs	1,431,004 sites	concatenation approach MLtree + IQtree
4d site SNPS	473,182 sites	concatenation approach MLtree + IQtree
200Kb window	2,973 trees	multispecies coalescent approach + ASTRAL
100Kb window	5,686 trees	multispecies coalescent approach + ASTRAL
50kb window	8,740 trees	multispecies coalescent approach + ASTRAL
25kb window	18,244 trees	multispecies coalescent approach + ASTRAL
10kb window	43,379 trees	multispecies coalescent approach + ASTRAL

Table S4 ASTRAL quartet score statistic

	Final quartet score	Final normalized quartet score
200kb window	61539082088	0.94474
100kb window	117057311494	0.94158
50kb window	180776271131	0.94241
25kb window	374156834032	0.93442
10kb window	874829880420	0.91887

Table S5 Comparison of demographic models analyzed with FASTSIMCOAL2.

Model	MaxEstLhood	MaxObsLhood	ΔL	AIC	ω
Model 1	-437328.901	-236962.506	200366.395	2013990	0
Model 2	-497695.907	-236962.506	260733.401	2292002	0
Model 3	-507076.217	-236962.506	270113.711	2335204	0
Model 4	-421507.404	-236962.506	184544.898	1941143	0
Model 5	-432940.711	-236962.506	195978.205	1993788	0
Model 6	-422898.564	-236962.506	185936.058	1947554	0
Model 7	-420327.258	-236962.506	183364.752	1935705	1
Model 8	-444611.903	-236962.506	207649.397	2047543	0

ΔL, MaxObsLhood-MaxEstLhood; AIC, Akaike's information criterion value; ω, AIC weight.

Table S6 Gene Ontology term enrichment in topo1 type genomic region

ID	Description	GO_Class	GeneRatio	BgRatio	pvalue	padj	Count
GO:0035556	intracellular signal transduction	BP	89/4972	159/13430	8.01E-07	0.000214	89
GO:0005216	ion channel activity	MF	81/4972	144/13430	1.89E-06	0.000253	81
GO:0006811	ion transport	BP	119/4972	230/13430	3.18E-06	0.000283	119
GO:0005096	GTPase activator activity	MF	36/4972	54/13430	9.12E-06	0.000609	36
GO:0004674	protein serine/threonine kinase activity	MF	49/4972	83/13430	3.62E-05	0.001934	49
GO:0043565	sequence-specific DNA binding	MF	99/4972	198/13430	0.000118	0.005238	99
GO:0004970	ionotropic glutamate receptor activity	MF	25/4972	38/13430	0.000295	0.011263	25
GO:0007165	signal transduction	BP	137/4972	294/13430	0.000425	0.014185	137
GO:0009190	cyclic nucleotide biosynthetic process	BP	20/4972	33/13430	0.004961	0.114333	20
GO:0016849	phosphorus-oxygen lyase activity	MF	20/4972	33/13430	0.004961	0.114333	20
GO:0006813	potassium ion transport	BP	16/4972	25/13430	0.005567	0.114333	16
GO:0019001	guanyl nucleotide binding	MF	16/4972	25/13430	0.005567	0.114333	16
GO:0031683	G-protein beta/gamma-subunit complex binding	MF	16/4972	25/13430	0.005567	0.114333	16
GO:0003700	DNA-binding transcription factor activity	MF	163/4972	378/13430	0.007789	0.148543	163
GO:0003713	transcription coactivator activity	MF	10/4972	14/13430	0.009474	0.168643	10
GO:0007219	Notch signaling pathway	BP	11/4972	17/13430	0.018922	0.280674	11
GO:0016779	nucleotidyltransferase activity	MF	9/4972	13/13430	0.018714	0.280674	9
GO:0016573	histone acetylation	BP	8/4972	11/13430	0.017709	0.280674	8
GO:0005230	extracellular ligand-gated ion channel activity	MF	29/4972	57/13430	0.022368	0.314334	29
GO:0003682	chromatin binding	MF	9/4972	14/13430	0.035345	0.400529	9
GO:0004402	histone acetyltransferase activity	MF	8/4972	12/13430	0.036003	0.400529	8
GO:0004714	transmembrane receptor protein tyrosine kinase activity	MF	8/4972	12/13430	0.036003	0.400529	8
GO:0007169	transmembrane receptor protein tyrosine kinase signaling pathway	BP	8/4972	12/13430	0.036003	0.400529	8
GO:0030145	manganese ion binding	MF	7/4972	10/13430	0.035688	0.400529	7
GO:0003824	catalytic activity	MF	75/4972	171/13430	0.038226	0.408253	75
GO:0051260	protein homooligomerization	BP	31/4972	64/13430	0.040209	0.412917	31

GO:0007018	microtubule-based movement	BP	33/4972	69/13430	0.04255	0.420771	33
GO:0003777	microtubule motor activity	MF	31/4972	65/13430	0.050256	0.443478	31
GO:0035091	phosphatidylinositol binding	MF	27/4972	56/13430	0.056432	0.443478	27
GO:0007154	cell communication	BP	15/4972	28/13430	0.054671	0.443478	15
GO:0009966	regulation of signal transduction	BP	15/4972	28/13430	0.054671	0.443478	15
GO:0018024	histone-lysine N-methyltransferase activity	MF	10/4972	17/13430	0.055833	0.443478	10
GO:0051015	actin filament binding	MF	10/4972	17/13430	0.055833	0.443478	10
GO:0004843	thiol-dependent ubiquitin-specific protease activity	MF	9/4972	15/13430	0.059795	0.443478	9
GO:0006820	anion transport	BP	9/4972	15/13430	0.059795	0.443478	9
GO:0043087	regulation of GTPase activity	BP	9/4972	15/13430	0.059795	0.443478	9
GO:0005089	Rho guanyl-nucleotide exchange factor activity	MF	45/4972	101/13430	0.071892	0.454908	45
GO:0035023	regulation of Rho protein signal transduction	BP	45/4972	101/13430	0.071892	0.454908	45
GO:0003774	motor activity	MF	26/4972	55/13430	0.07667	0.454908	26
GO:0016459	myosin complex	CC	26/4972	55/13430	0.07667	0.454908	26
GO:0006351	transcription, DNA-templated	BP	21/4972	43/13430	0.075243	0.454908	21
GO:0004386	helicase activity	MF	12/4972	22/13430	0.071107	0.454908	12
GO:0006397	mRNA processing	BP	12/4972	22/13430	0.071107	0.454908	12
GO:0005886	plasma membrane	CC	7/4972	11/13430	0.067152	0.454908	7
GO:0031625	ubiquitin protein ligase binding	MF	7/4972	11/13430	0.067152	0.454908	7
GO:0008270	zinc ion binding	MF	190/4972	473/13430	0.082043	0.476209	190

Table S7 KEGG pathways enrichment in topo1 type genomic region

ID	Description	GeneRatio	BgRatio	pvalue	padj	Count
map04713	Circadian entrainment	95/2978	169/7961	4.08E-07	0.000106	95
map04520	Adherens junction	74/2978	135/7961	2.54E-05	0.003299	74
map04015	Rap1 signaling pathway	156/2978	333/7961	0.000202	0.017512	156
map04720	Long-term potentiation	55/2978	104/7961	0.000873	0.056775	55
map00720	Carbon fixation pathways in prokaryotes	14/2978	19/7961	0.001429	0.07431	14
map04728	Dopaminergic synapse	99/2978	209/7961	0.001813	0.078556	99
map04310	Wnt signaling pathway	99/2978	210/7961	0.002189	0.081292	99
map04727	GABAergic synapse	68/2978	138/7961	0.002712	0.088139	68
map04724	Glutamatergic synapse	94/2978	201/7961	0.003746	0.108227	94
map04730	Long-term depression	47/2978	92/7961	0.00491	0.127655	47
map04151	PI3K-Akt signaling pathway	191/2978	444/7961	0.007197	0.151419	191
map00230	Purine metabolism	84/2978	181/7961	0.007571	0.151419	84
map04120	Ubiquitin mediated proteolysis	73/2978	154/7961	0.006633	0.151419	73
map04010	MAPK signaling pathway	174/2978	404/7961	0.009505	0.172774	174
map04916	Melanogenesis	70/2978	149/7961	0.009968	0.172774	70
map04360	Axon guidance	134/2978	307/7961	0.012926	0.210049	134
map04024	cAMP signaling pathway	156/2978	366/7961	0.02048	0.24277	156
map04014	Ras signaling pathway	128/2978	294/7961	0.016293	0.24277	128
map04022	cGMP-PKG signaling pathway	116/2978	267/7961	0.022932	0.24277	116
map04390	Hippo signaling pathway	99/2978	225/7961	0.023343	0.24277	99
map04725	Cholinergic synapse	85/2978	190/7961	0.021616	0.24277	85
map04540	Gap junction	62/2978	134/7961	0.02122	0.24277	62
map04340	Hedgehog signaling pathway	32/2978	63/7961	0.020268	0.24277	32
map00770	Pantothenate and CoA biosynthesis	10/2978	15/7961	0.02055	0.24277	10
map04711	Circadian rhythm - fly	9/2978	13/7961	0.020097	0.24277	9
map04013	MAPK signaling pathway - fly	50/2978	106/7961	0.024368	0.243678	50
map04910	Insulin signaling pathway	78/2978	175/7961	0.02954	0.284462	78

map04960	Aldosterone-regulated sodium reabsorption	24/2978	47/7961	0.03839	0.35648	24
map04726	Serotonergic synapse	73/2978	165/7961	0.040856	0.366295	73
map04924	Renin secretion	51/2978	112/7961	0.046493	0.40294	51

Table S8 D-statistic analysis results.

Table S9 The QuIBL analysis results.

See attachment xlsx file
